# Current Research on the Bioprospection of Linear Diterpenes from *Bifurcaria bifurcata*: From Extraction Methodologies to Possible Applications

**DOI:** 10.3390/md17100556

**Published:** 2019-09-28

**Authors:** Adriana C.S. Pais, Jorge A. Saraiva, Sílvia M. Rocha, Armando J.D. Silvestre, Sónia A.O. Santos

**Affiliations:** 1 CICECO-Aveiro Institute of Materials, Chemistry Department, University of Aveiro, 3810-193 Aveiro, Portugal; 2 QOPNA/LAQV & REQUIMTE, Chemistry Department, University of Aveiro, 3810-193 Aveiro, Portugal; jorgesaraiva@ua.pt (J.A.S.); smrocha@ua.pt (S.M.R.)

**Keywords:** *Bifurcaria bifurcata*, linear diterpenes, extraction, identification, biological activities, macroalgae, high value applications

## Abstract

Marine resources are considered as a very promising source of bioactive molecules, and macroalgae in particular have gained special attention, due to their structurally diverse composition. Particular interest has been devoted to the brown macroalga *Bifurcaria bifurcata*, due to their abundance in bioactive linear diterpenes. In this appraisal, a thorough review concerning the methodologies used in the extraction, fractionation, and identification of diterpenes from *B. bifurcata* is provided and discussed in detail. An exhaustive compilation of the mass spectra and nuclear magnetic resonance (NMR) data are also provided. The in vitro and *in chemico* assays already performed to assess different biological activities attributed to *B. bifurcata* diterpenes are also reviewed, emphasizing the use of isolated components, enriched fractions, or crude extracts. The associated major strengths and challenges for the exploitation of *B. bifurcata* diterpenes for high-value applications are critically discussed.

## 1. Introduction

Marine resources have been seen as a promising source of added value molecules, an alternative of finite fossil resources, allowing for the boost of concepts such as biorefinery, circular economy, and blue economy [1,2,3]. In recent years, an increase in marine biotechnology investments has been observed around the world, with macroalgae being an object of particular interest, mainly due to their high productivity, wide diversity, and heterogeneous composition, notably with a high abundance of bioactive compounds [4]. In fact, besides the high content of polysaccharides, macroalgae have shown to be particularly rich in secondary metabolites with a wide range of biological activities, which can be exploited as functional ingredients, or in cosmetic and pharmaceutical formulations [4,5]. However, macroalgae metabolism, and consequently composition, is modulated by, among other factors, salinity, temperature, pressure, sunlight, geographic origin, and season of collection [6,7]. 

Macroalgae are ecologically and commercially important, being significant primary producers in oceanic aquatic foods chains [6]. These marine resources have been recognized to be a valuable source of polysaccharides, minerals, polyunsaturated fatty acids and vitamins, and, in some species, of phenolic compounds and terpenes [6,8,9].

Special attention has been devoted to brown macroalgae in particular, due to the presence of specific components, such as fucoidan, phlorotannins, or even fucoxanthin, for which promising bioactivities have been described, namely antitumor, antioxidant, and antihypertensive activities, among others [10,11,12].

Notwithstanding, brown macroalgae have attracted an increasing attention due to the presence of other compounds, such as some linear diterpenes commonly found in the Sargassaceae family, and particularly in *Bifurcaria bifurcata* species [13]. Compared to their cyclic congeners, these are quite rare in nature. In addition, a wide variety of promising biological activities have been attributed to these components. Although a detailed revision of the different linear diterpenes already identified in *B. bifurcata* has been previously reported [14], an overview of the *B. bifurcata* diterpenes’ properties together with information about how these components can be extracted and identified is needed in order to boost their exploitation. In this vein, the purpose of this paper is to review the different extraction and characterization methodologies used to extract and identify linear diterpenes from *B. bifurcata* as well as the current findings on their biological properties. The major challenges and strengths for the exploitation of this fraction of *B. bifurcata* are also discussed.

## 2. *Bifurcaria bifurcata* General Characteristics and Linear Diterpenes Chemical Families

*B. bifurcata* is a brown macroalga, and due to its morphology it is classified as a cylindrical species [15] and can be scientifically classified as follows [16]:

- Empire: Eukaryota
- Kingdom: Chromista
- Phylum: Ochrophyta
- Class: Phaeophyceae
- Subclass: Fucophycidae
- Order: Fucales
- Family: Sargassaceae
- Genus: *Bifurcaria*

**Figure 1:**
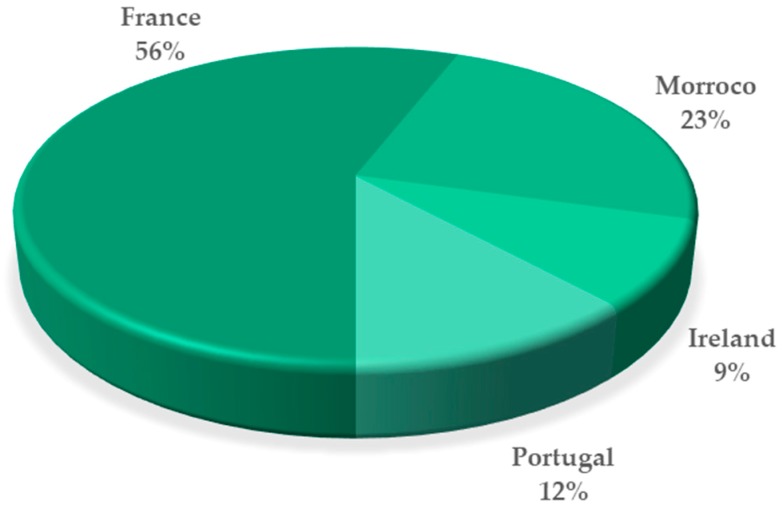
Distribution, in percentage, of the studies on *B. bifurcata* linear diterpenes and/or on related biological activities, according to the sampling location. The percentages presented in this graphic were determined considering the number of samples of each geographical location in all published studies here reviewed. (Analyzing Institute for Scientific Information (ISI) Web of Knowledge, keywords: *Bifurcaria bifurcata,* diterpenes; timespan: 1980–2019).

This brown macroalga lives in rock pools on the lower and middle tidal, needing shores for its settlement, and is distributed along the coast of the Northern Atlantic, between Morocco and Northwestern Ireland [17]. About 56% of the published (and here reviewed) studies concerning the linear diterpenes fraction of *B. bifurcata* use samples from the coast of France (Figure 1), which may be associated with the high abundance of this macroalga on that area [8,17,18,19,20,21,22,23,24,25,26,27,28,29,30,31,32,33,34,35,36,37].

Additionally, all studies reviewed here have used wild samples of *B. bifurcata*, with the exception of the work of Santos et al. [38], in which *B. bifurcata* was collected from the Portuguese coast and cultivated in a land-based aquaculture system for three weeks at constant controlled conditions. Concerning the different sampling locals as well as the exposure to different environmental factors, it is expected that this variety of *B. bifurcata* presents diverse compositions.

### Linear Diterpenes Families of B. bifurcata

Phaeophyceae’s class members are, in general, widely known for their ability to produce a large variety of terpenoids [4]. Terpene’s chemical structures can be considered as being formed by multiple isoprene (C_5_H_8_) units (although being biosynthesized from isoprene derived activated precursors, as described below), and are relatively complex and extremely diverse, since they can have a linear, monocyclic, or bicyclic structure, among others, with different unsaturation degrees and patterns, as well as different levels of oxygenation [39,40]. This diversity can result in different chemical and biological properties [41]. Terpenes may be classified by the number of isoprene units that integrate the molecule, being grouped into hemi- (C_5_), mono- (C_10_), sesqui- (C_15_), di- (C_20_), sester- (C_25_), tri- (C_30_), tetra- (C_40_), and polyterpenes, depending on the number of isoprene units [39,40].

**Figure 2:**
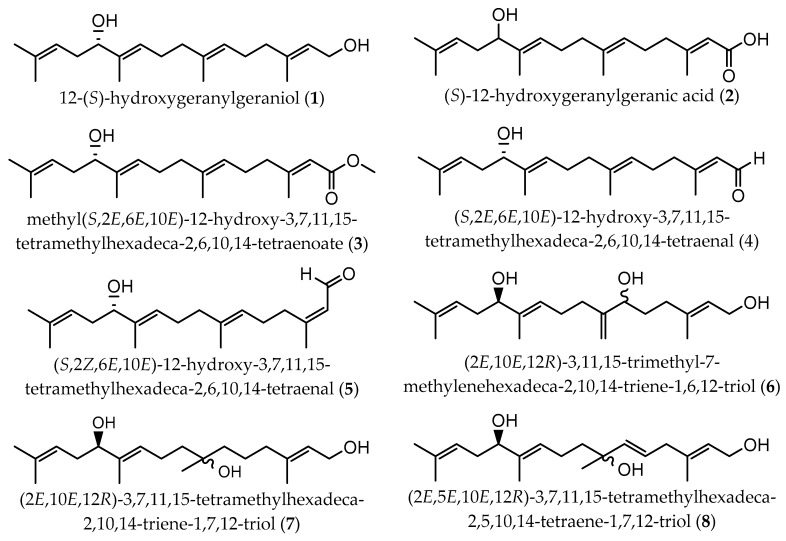
Chemical structures of acyclic diterpenes identified in *B. bifurcata*, belonging to family A.

**Figure 3:**
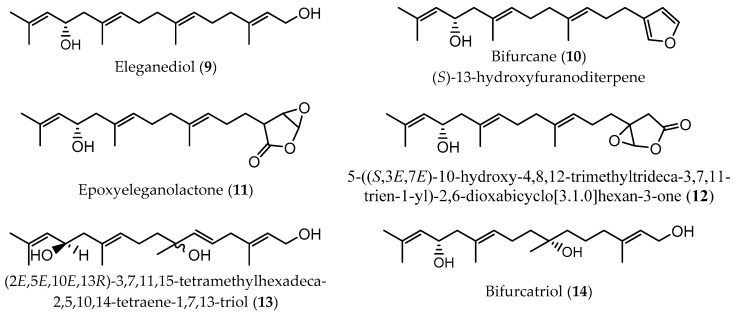
Chemical structures of acyclic diterpenes identified in *B. bifurcata*, belonging to family B1.

**Figure 4:**
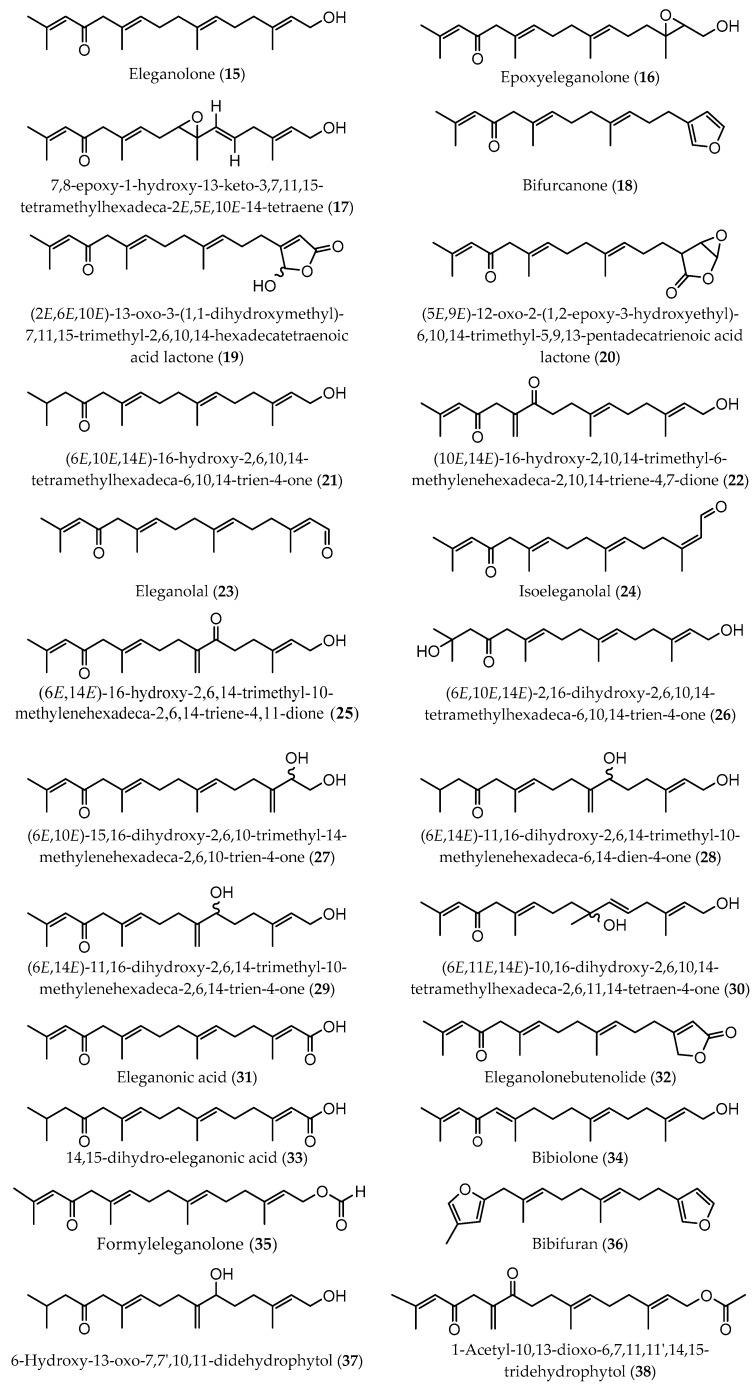
Chemical structures of acyclic diterpenes identified in *B. bifurcata*, belonging to subfamily B2.

**Figure 5:**
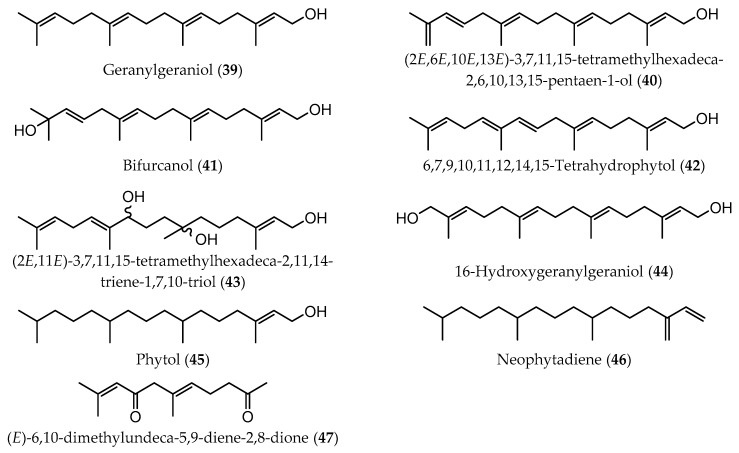
Chemical structures of acyclic diterpenes identified in *B. bifurcata*, belonging to family C.

Meroditerpenes (which correspond to a complex of terpenic and aromatic parts, and can be subdivided into linear, cyclic, and rearranged terpenoids) and linear diterpenes are the major terpenoids described in Sargassaceae family [40]. Linear (or acyclic) diterpenes, like all isoprenoids, are biosynthesized from isopentenyl diphosphate (IPP) and dimethylallyl diphosphate (DMAPP), which are formed via classical mevalonate (MVA) pathway from the condensation of acetyl-CoA or via 1-deoxy-D-xylulose 5-phosphate/2-C-methyl-D-erythritol 4-phosphate (DOXP/MEP) pathway, from the intermediates pyruvate and D-glyceraldehyde-3-phosphate (GAP) [42]. These linear terpenes are made up of a C_16_ backbone with four double bonds and generally with five non-substituted methylene groups. According to the main chemical characteristics, linear diterpenes are arranged in three families: A (C-12 oxidized compounds) (Figure 2); B (two subfamilies: on the one hand, B1 corresponds to C-13 oxidized molecules bearing an OH group, while on the other hand B2 are compounds with a ketone functionality at C-13 position) (Figure 3 and Figure 4, respectively); and C (which are the non C-12/C-13 oxidized compounds) (Figure 5). These four groups of compounds (A, B1, B2, and C) have metabolic precursors, which are 12-(S)-hydroxygeranylgeraniol (bifurcadiol) (**1**), eleganediol ((S)-13-hydroxygeranylgeraniol) (**9**), eleganolone (13-oxogeranylgeraniol or (S)-13-ketogeranylgeraniol) (**15**), and geranylgeraniol (**39**), respectively [14].

Linear diterpenes belonging to these 4 groups, have already been found in samples of *B. bifurcata* from different geographical origins and exposed to different external factors, which, as mentioned above, have a high influence on the macroalgae’s chemical composition [6,7]. The detailed list of the linear diterpenes (and their content when available) already identified in *B. bifurcata* is depicted in Table 1. It must be highlighted that, due to the variation of sampling origin, season of collection, and thus different abiotic factors, together with the different extraction and characterization methodologies used by the different authors, it is not possible to establish correlations between these factors, conditions, or methodologies and the linear diterpenes profile detected.

Biard et al. reported for the first time the presence of linear diterpenes in *B. bifurcata* extracts, namely eleganolone (**15**), the major component (2% of dry weight (dw)), and two derivatives (**16** (0.06% of dw) and **9** (0.28% of dw)) [19,20]. The eleganolone (**15**) content in two samples from Brittany, France was also determined by Maréchal et al. and Hellio et al., which accounted 0.06% and 0.47% of dw, respectively. This difference could be related with the effect of several external factors, including season and sampling local, among others [23,36].

Le Lann et al. identified four distinct chemical profiles in extracts of *B. bifurcata*, collected during summer 2009 and winter 2010, from six regions along the Western coast of Britany, France, evidencing the high dependence of diterpenes composition on the sampling site and environmental factors. Eleganolone (**15**) was also identified as the major compound in both winter and summer seasons from these macroalgae samples, whereas bifurcanone (**18**) was only found during winter. The eleganediol (**9**), bifurcane (**10**), eleganolone (**15**), and bifurcanone (**18**) contents were shown to be also influenced by season and environmental factors [17].

In fact, eleganediol (**9**) [19,23,24,26,28,29,31,33,36,37], bifurcane (**10)** [29,30,31,36], and eleganolone (**15**) [20,21,22,23,24,26,27,28,30,31,36,37] have been described in several studies concerning *B. bifurcata* from France, together with other linear diterpenes, namely formyleleganolone (**35**) and bibifuran (**36**) [34]. The highest eleganediol (**9**) and bifurcane (**10**) contents (1.12% and 0.37% of dw, respectively) were described by Maréchal et al. [36]. Furthermore, 12-(*S*)-hydroxygeranylgeraniol (**1**) was only found in samples of *B. bifurcata* collected from Morocco, corresponding to 0.71% of dw studied by Hellio et al. [23,43,44,45,46].

In addition to bifurcane (**10**) and bibifuran (**36**), other *B. bifurcata* constituents are characterized by the presence of a furan ring in their structure, such as bifurcanone (**18**) and eleganolonebutenolide (**32**), which belong to B2 subfamily (C-13 oxidized molecule bearing an alcohol). Epoxyeleganolactone (**11**), which is a diterpene from B1 subfamily (with a ketone functionality at C-13 position), presents a C-2 epoxylactone [28,29,30].

Some of the identified compounds have an acidic character due to the presence of a carboxylic group at C-1, such as the case of (*S*)-12-hydroxygeranylgeranic (**2**), eleganonic (**31**), and 14,15-dihydro-eleganonic acids (**33**) [30,34,43,47]. Semmak et al. reported the highest (*S*)-12-hydroxygeranylgeranic acid (**2**) content on *B. bifurcaria* from Morocco, which accounted 0.17% of dw [47].

Culioli et al. reported for the first time the presence of (*E*)-6,10-dimethylundeca-5,9-diene-2,8-dione (**47**) (0.01% of dw) in *B. bifurcata*, which resulted from the oxidative cleavage of the C-6 double bond of eleganolone (**15**) [33]. In addition, two pairs of isomers were also isolated from *B. bifurcata*, namely (*S*,2*E*,6*E*,10*E*)-12-hydroxy-3,7,11,15-tetramethylhexadeca-2,6,10,14-tetraenal (**4**) and (*S*,2Z,6*E*,10*E*)-12-hydroxy-3,7,11,15-tetramethylhexadeca-2,6,10,14-tetraenal (**5**), and eleganolal (**23**) (0.12% of dw) and isoeleganolal (**24**) (0.08% of dw) [32,46].

Several diterpenes were identified and quantified in a detailed study of the lipophilic fraction of a short-term cultivated *B. bifurcata,* collected in May, from Ria de Aveiro, Portugal, namely 6-hydroxy-13-oxo-7,7’,10,11-didehydrophytol (**37**) (0.06% of dw), 1-acetyl-10,13-dioxo-6,7,11,11’,14,15-tridehydrophytol (**38**) (0.10% of dw), geranylgeraniol (**39**) (0.01% of dw), 6,7,9,10,11,12,14,15-tetrahydrophytol (**42**) (0.01% of dw), phytol (**45**) (0.003% of dw), and neophytadiene (**46**) (0.01% of dw) [38]. Compounds **37**, **38**, **45**, and **46** were identified for the first time as constituents of this macroalga species, whereas compounds **39** [23,31,36,37] and **42** [46] had already been identified in samples from different geographical origins, namely from Morocco and France. A higher geranylgeraniol (**39**) content was verified by Hellio et al. in *B. bifurcata* from Morocco (0.12% of dw) than those determined by Culioli et al. and Maréchal et al. (0.01% and 0.02% of dw, respectively) in samples from Brittany, France and from Portugal [23,32,36,38].

## 3. Characterization of Linear Diterpenes from *B. bifurcata*

The bioprospection of linear diterpenes from *B. bifurcata* involves a sequential approach, including extraction, fractionation, identification, and quantification steps. Hence, in the following subchapters the methodologies already used to extract, fractionate, and characterize these bioactive compounds will be described.

### 3.1. Extraction and Fractionation Methodologies

Sample drying and milling are the first steps in the analysis of active compounds from plant raw materials. Macroalgae sample drying is a preliminary step that is often carried out prior to milling and extraction steps, and could be done by air drying (in the shade or not) [23,24,26,31,36,37,44,46,49,52] or by freeze-drying [8,19,20,21,22,27,28,29,30,34,35,38,43,50,51,53,54,55]. Notwithstanding, Semmak et al. have obtained linear diterpenes from freshly collected *B. bifurcata* [47]. The sample drying step is very important, especially when nonpolar extraction solvents are used, in order to avoid moisture contents that can diminish the extraction efficiency. Contrarily, when polar solvents [methanol (MeOH), ethanol (EtOH), ethyl acetate (EtOAc), among others] or solvent mixtures [hexane/acetone, hexane/acetonitrile (MeCN), etc.] are used, the use of wet samples can be considered [56]. Notwithstanding, it must be highlighted that when freshly collected macroalga is used, the moisture content is uncontrolled, which, consequently, compromises the extraction reproducibility.

The milling step could also have an important influence in the extraction, specifically in the compounds diffusion from the matrix to the solvent, since by decreasing the particles size, the surface area increases, improving the compounds diffusion [56,57]. Thus, the biomass is often ground prior to extraction [8,21,22,23,24,27,29,31,34,35,36,37,38,43,44,45,46,51,54,55,58,59]. Extraction is considered one of the most relevant steps, being a mass transport phenomenon, where components are transferred from the plant matrix to a solvent up to their equilibrium concentration [56]. The extraction efficacy is affected by several factors, such as the technique used to the sample drying, the particle size, the extraction solvent (or mixtures), temperature, and extraction time [60]. The different extraction conditions that have been applied to extract linear diterpenes from *B. bifurcata* are summarized in Table 1. 

It should be highlighted that the effect of different solvents, extraction time, or even temperature used is difficult to critically compare, on one hand due to the different geographical origin or season of macroalgae sampling in each study, and, on the other hand, by the lack of accurate diterpenes quantification in many studies. Notwithstanding, an overview of the most commonly applied conditions will be reported and discussed. 

Conventional solid–liquid extraction with continuous stirring (maceration) has been amongst the most used extraction methodology to obtain linear diterpenes from *B. bifurcata* [19,20,21,22,23,24,26,27,28,29,30,31,32,33,34,36,37,43,44,45,46,47,48,49,50,51]. The extraction yields (EY) obtained by maceration ranged from 0.95% to 9.06% (*w*/*w*) [21,23,24,27,28,29,31,34,36,37,43,44,45,46,47,49,50,51]. This wide range could be a consequence of the different solvents used and/or the different geographical origins. The conventional Soxhlet extraction has also been shown to be efficient in extracting these compounds from *B. bifurcata* (EY of 3.92% ± 0.09% (*w*/*w*)) [38]. Diethyl ether (Et_2_O) and EtOAc were the most used solvents, resulting in variable EYs, 1.65%–3.67% and 1.70%–4.80% (*w*/*w*), respectively [19,20,21,22,23,26,27,28,29,30,31,32,33,34,36,43,46,52]. Moreau et al., Ortalo-Magné et al., Semmak et al*.,* among others, selected a mixture of chloroform–methanol (CHCl_3_:MeOH) to extract different linear diterpenes from *B. bifurcata*, resulting in EYs from 2.63% to 6.92% (*w*/*w*) [24,37,44,47,49], while other authors have selected different mixtures of solvents (such as EtOH:CHCl_3_, leading to EY of 1.52% (*w*/*w*)) [45]. 

Despite being a very important variable, most of studies did not specify the extraction time applied. Concerning the Soxhlet extraction with dichloromethane (DCM), an extraction time of 9 h was reported to be effective in the extraction of linear diterpenes from *B. bifurcata* [38]. Similarly, Combaut et al. carried out three consecutive solid–liquid extractions, also using Et_2_O with an extraction time of 3 h, resulting in a EY of 3.67% (*w*/*w*) [27]. However, considerably higher extraction times were also used, such as in the study published by Maréchal et al., where an Et_2_O solid–liquid extraction during 48 h also proved to be efficient in obtaining *B. bifurcata* linear diterpenes, with extraction yields ranging from 2.2% to 2.9% (*w*/*w*) [36]. 

Most of the diterpenes described in Table 1 were extracted from *B. bifurcata* at room temperature [21,22,23,24,27,29,31,32,34,35,36,37,43,44,46,50,59,61]. It is not clear from the reported data to what extent *B. bifurcata* diterpenes are thermosensitive, however their extraction by Soxhlet have been shown to be successful. Soxhlet extraction with DCM, at temperatures close to 40 °C, allowed researchers to extract about 1.9 g of diterpenes kg^−1^ of macroalga dw [38].

It should be highlighted that most of the studies concerning the extraction of diterpenes from *B. bifurcata* were carried out with analytical and bioprospection purposes, justifying the use of conventional methodologies with organic and often hazardous solvents. However, the exploitation of these bioactive components to high-value applications deserves the development and optimization of environmentally friendly and sustainable extraction approaches. Actually, the extraction of diterpenes from this macroalga species has been characterized by long operating times, and therefore high energetic consumption and the use of organic solvents, for which toxicity might compromise the exploitation of these fraction/extracts in food, nutraceutical, or pharmaceutical fields. New and promising environmental friendly extraction techniques have been developed to extract bioactive components from biomass, for example, ultrasound assisted extraction, microwave-assisted extraction, high pressure extraction, among others [57,60]. Due to the small amount of organic solvents used or even the possibility of their replacement by water, they are recognized as green (or eco-friendly) technologies [60,62]. In addition, these emerging technologies aim to shorten the extraction time, intensifying the mass transfer process, as well as to increase the extraction yields, resulting also in higher extracts quality and reducing the energy consumption [63]. Therefore the exploitation of these emerging technologies should allow researchers to widen the field of applications of *B. bifurcata* linear diterpenes fractions/extracts.

Fractionation and, in some cases, purification steps have been performed after extraction. Some authors have obtained enriched fractions by applying successive extraction methodologies [37,54,55,61] or even isolated compounds by fractionation with column chromatography (CC), followed, in most cases, by HPLC fractionation [20,23,24,27,29,30,31,34,36,43,44,45,46,47,49,51]. 

Usually, “bio-guided fractionation” has been applied as a strategy to isolate the main constituent of the most active fraction [21,28,35]. Although, Nardella et al. applied a new strategy to accelerate the discovery of new bioactive compounds, combining two-dimensional (2D) NMR analysis with high performance liquid chromatography with diode array detection coupled to mass spectrometry and solid phase extraction (HPLC-DAD-MS-SPE-NMR) to identify eleganolone (**15**). The authors called this new strategy “pharmacophoric deconvolution”, which proved to be three times faster and to require less starting raw material than “bio-guided fractionation” [22]. 

### 3.2. Instrumental Analysis: Identification and Quantification

The identification of linear diterpenes from *B. bifurcata* has frequently been performed using spectroscopic techniques, such as infrared spectroscopy (IR), ultraviolet spectroscopy (UV), one (1D) and 2D (^1^H and ^13^C) NMR and high resolution mass spectrometry (HRMS) [19,20,24,27,28,29,30,31,32,33,34,36,43,44,45,46,47,49,51]. In fact, both NMR and mass spectrometry (MS) analysis can be expeditious ways to identify these components, since unique spectroscopic profiles can be recorded for each compound. 

For example, Maréchal et al. obtained an Et_2_O extract, with 2.2% to 2.9% of algal dw, and identified and quantified eleganediol (**9**) through IR, UV, HRMS, and 1D and 2D NMR [36]. Taking into account the MS data obtained by Biard et al., the mass spectrum of compound **9** presents a molecular ion [M]^+^ at *m*/*z* 306 representing the chemical formula C_20_H_34_O_2_ and also other characteristic product ions, namely [M − H_2_O]^+^ at *m*/*z* 288 [19]. Additionally, they carried out ^13^C NMR analysis for its identification [19]. Valls et al. have also identified **9** by ^1^H (360 MHz, deuterated chloroform (CDCl_3_); 200 MHz, deuterated benzene (C_6_D_6_)), ^13^C (90 MHz, CDCl_3_; 50 MHz, C_6_D_6_), and distortionless enhancement by polarization transfer (DEPT) NMR [29].

Culioli et al. identified the chemical formula of methyl(*S,*2*E*,6*E*,10E)-12-hydroxy-3,7,11,15-tetramethylhexadeca-2,6,10,14-tetraenoate (**3**) (C_21_H_34_O_3_), (*S*,2*E*,6*E*,10*E*)-12-hydroxy-3,7,11,15-tetramethylhexadeca-2,6,10,14-tetraenal (**4**) (C_20_H_32_O_2_), (*S*,2Z,6*E*,10*E*)-12-hydroxy-3,7,11,15-tetramethylhexadeca-2,6,10,14-tetraenal (**5**) (C_20_H_32_O_2_), and 6,7,9,10,11,12,14,15-tetrahydrophytol (**42**) (C_20_H_32_O) by HRMS (Table S1), observing the molecular ions [M]^+^ at *m/z* 334.2506, 304.2408, 304.2410, and 288.2450, respectively [46]. Furthermore, electron ionization mass spectrometry (EIMS) (70 eV) analysis was carried out to complement the identification, in which the molecular ions [M]^+^ were confirmed. In addition, other characteristic product ions were also observed, for instance, in the MS spectrum of compound **3** the presence of the ions at *m*/*z* 316 [M − H_2_O]^+^, 265, 233, 215, 187,137, 123, 107, 93, 81, 69, 55 was verified [46].

Eleganolone (**15**) was identified by Gallé et al. [21] through high resolution electron ionization mass spectrometry (HREIMS) and ^1^H (400 MHz, CDCl_3_), ^13^C (100 MHz, CDCl_3_), and DEPT NMR, by comparison with the data described in literature [20,31]. NMR spectral data are described in Tables S2 and S3. The HREIMS of compound **15** present an ion peak for the sodium adduct ([M + Na]^+^) at *m*/*z* 327.23193, indicating the molecular formula C_20_H_32_O_2_ [21]. 

Smyrniotopoulos et al. identified a new linear diterpene, bifurcatriol (**14**), from an Irish sample of *B. bifurcata*, using the spectroscopic techniques mentioned above (IR, 1D and 2D NMR, HRMS) and also experimental and computational vibrational circular dichroism (VCD) spectroscopy, which was used to determine the structure and absolute configuration of the compound [50]. Taking into account its HRMS, the ion [M + Na]^+^ was observed at *m*/*z* 347.2559, being assigned to the molecular formula C_20_H_36_O_3_ [50]. 

Furthermore, chromatographic systems coupled to spectroscopic techniques, such as gas chromatography-mass spectrometry (GC-MS) have been also applied, with the advantage to simultaneously allowing identification and quantification [38]. Santos et al. identified and quantified the linear diterpenes present in a DCM extract from *B. bifurcata*, accounting 0.189% ± 0.013% of dw, which corresponded to about 57% of the total amount of lipophilic compounds detected [38]. The direct GC-MS analysis of the extract, using a DB-1 column (Agilent) and without any previous fractionation or derivatization, allowed them to identify six linear diterpenes. Hereof, this study stands out from the others here reviewed, which involved mostly NMR analysis, often accomplished by previous fractionation and/or purification steps. It must be highlighted the mass spectrum obtained for neophytadiene (**46**), in which the molecular ion [M]^+^ at *m*/*z* 278, and also some major product ions at *m*/*z* 43 ([C_3_H_7_]^+^), 57 ([C_4_H_9_]^+^), 68, 82, 95 ([C_7_H_11_]^+^), 109, and 123 ([C_9_H_15_]^+^), were observed. In the same way, geranylgeraniol (**39**) mass spectrum presented the molecular ion [M]^+^ at *m*/*z* 290, and characteristic products ions at *m*/*z* 69 ([C_5_H_9_]^+^), 81 ([C_6_H_9_]^+^), 121 ([C_11_H_19_]^+^), 272 ([M − H_2_O]^+^)[38].

A more detailed discussion of the spectroscopic features of these compounds is beyond the scope of the present review, however its availability in a systematized way is undoubtedly important for the readers in the field. Therefore, an exhaustive compilation of NMR and MS data for linear diterpenes from *B. bifurcata* is systematized in supplementary Tables S1, S2, and S3.

## 4. Biological Activities of *B. bifurcata* Diterpenes-Enriched Extracts: In vitro and *In Chemico* Assays 

The interest in *B. bifurcata* linear diterpenes-enriched extracts has increased in recent years due to the vast range of biological activities already associated with these compounds. Despite being very rare in nature, they are present in high amounts in *B. bifurcata* lipidic extracts, as shown above. An overview of the biological activities already reported for *B. bifurcata* linear diterpenes-enriched extracts or purified components is present in Table 2 (studies involving isolated diterpenes from *B. bifurcata* and those involving *B. bifurcata* extracts previously found to be rich in linear diterpenes were considered).

Most of the studies have evaluated the biological activities of *B. bifurcata* linear diterpenes and extracts through in vitro or *in chemico* assays. Therefore, the future development of in vivo assays to corroborate these results may be crucial to their exploitation for high-value applications. 

### 4.1. Antimicrobial Activity

Biard et al. firstly reported that the Et_2_O extract of *B. bifurcata* showed antimicrobial activity against *Mycobacterium smegmatis* [20] Subsequently, they proceeded to the isolation of the major compound, namely eleganolone (**15**), which also inhibited the bacteria growth at 75 μg mL^−1^ (expressed as minimal inhibitory concentration (MIC)), as well as that of *Bacillus subtilis* (MIC = 2.5 mg mL^−1^), *Mycobacterium aquae* (MIC = 400 μg mL^−1^), *Mycobacterium ranae* (MIC = 100 μg mL^−1^), *Mycobacterium xenoqui* (MIC = 200 μg mL^−1^), and *Mycobacterium avium* (MIC = 100 μg mL^−1^) [20]. Hellio et al. also reported the antibacterial activity of eleganediol (**9**) and eleganolone (**15**) against a strain of *Bacillus sp*. (MIC = 8 μg mL^−1^) [23].

Furthermore, Hellio et al. reported the antimicrobial activity of two eleganolone (**15**) derivatives, ((10*E*,14*E*)-16-hydroxy-2,10,14-trimethyl-6-methylenehexadeca-2,10,14-triene-4,7-dione (**22**) and (6E,14E)-16-hydroxy-2,6,14-trimethyl-10-methylenehexadeca-2,6,14-triene-4,11-dione (**25**), against a gram-positive bacteria, namely *Bacillus sp*. (MIC = 8 μg mL^−1^), as well as against marine fungi, namely *Corollospora maritima, Lulworthia* sp., and *Dendryphiella salina*, MIC = 8 μg mL^−1^ [23].

Santos et al. demonstrated that a DCM *B. bifurcata* extract rich in linear diterpenes (48.29 mg g^−1^ of extract) exhibited antibacterial activity against both gram-positive (namely, *Staphylococcus aureus* ATCC®6538 (MIC = 1024 μg mL^−1^) and ATCC®43300 (MIC = 2048 μg mL^−1^)) and gram-negative (in particular, *Escherichia coli* ATCC®25922 (MIC = 2048 μg mL^−1^)) bacteria. In addition, this extract was shown to reinforce the antimicrobial activity of several antibiotics. The combination of the extract with gentamicin or tetracycline resulted in a severe decrease of the antibiotic MIC against the three strains. Rifampicin MIC also decreased about 87% and 50% in the presence of the extract toward *Staphylococcus aureus* ATCC®43300 and *Escherichia coli* ATCC®25922, respectively. Thus, this synergism of *B. bifurcata* extracts with antibiotics could be further exploited in a way to overcome the antibiotic-resistant bacteria strains, which is a serious public health problem [38]. 

Alves et al. studied the antibacterial activity (expressed in zone of inhibition (ZI)) of MeOH and DCM extracts of *B. bifurcata*. Both extracts were revealed to be active against *Escherichia coli* ATCC 10536 (ZI: 7.0 ± 0.0 mm and 8.3 ± 0.6 mm for MeOH and DCM extracts, respectively), while MeOH extract was also shown to inhibit *Staphylococcus aureus* ATCC 25923 (7.0 ± 0 mm ZI) [55]. The MeOH extract also presented antibacterial activity against *Bacillus subtilis* ATCC 6633 (6.7 ± 0.6 mm ZI) and *Escherichia coli* ATCC 25922 (6.7 ± 0.6 mm ZI) [55]. In fact, antimicrobial activity of both MeOH and DCM *B. bifurcata* extracts was also demonstrated against another gram-negative bacteria, *Pseudomonas aeruginosa* ATCC 27853 (11.3 ± 1.5 mm ZI and 8.3 ± 1.2 mm ZI, respectively), and toward *Saccharomyces cerevisiae* ATCC 9763, which was expressed in the inhibitory concentration of the extract required to decrease microbial concentration by 50% (IC_50_), IC_50_ = 26.68 μg mL^−1^ and IC_50_ = 17.06 μg mL^−1^, for MeOH and DCM extracts, respectively [55]. According to these results, a higher potential antibacterial activity against *Pseudomonas aeruginosa* ATCC 27853 in the MeOH extract was observed while the DCM extract showed a higher antifungal activity against *Saccharomyces cerevisiae* ATCC 9763 [55].

Antiprotozoal activity has been one of the most studied biological activities for linear diterpenes from *B. bifurcata.* Protozoal infections, in particular malaria, leishmaniosis, and sleeping sickness, among others, are fatal diseases [35]. Recently, Smyrniotopoulos et al. proved the antiprotozoal activity of bifurcatriol (**14**), obtained after several purification steps [50]. This linear diterpene also revealed antimalarial activity toward a resistant K1 strain of the malaria parasite, *Plasmodium falciparum* (IC_50_ = 0.65 ± 0.05 μg mL^−1^), with a good selectivity, and protozoal activity against *Trypanosoma brucei rhodesiense* (IC_50_ = 11.8 ± 0.01 μg mL^−1^)*, Trypanosoma cruzi* (IC_50_ = 47.8 ± 0.59 μg mL^−1^), and *Leishmania donovani* (IC_50_ = 18.8 ± 0.12 μg mL^−1^) [50]. After the evaluation of the antiprotozoal activity of an EtOAc extract, through a bio-guided fractionation, Gallé et al. proved that eleganolone (**15**) was responsible for the antimalarial (or anti-plasmodial) activity of the extract toward *Plasmodium falciparum* (IC_50_ = 2.6 μg mL^−1^), with a good selectivity index (SI = 21.6). However, this component presented lower trypanocidal activity against *Trypanosoma brucei rhodesiense* (IC_50_ = 13.7 μg mL^−1^) and *Trypanosoma cruzi* (IC_50_ = 17.7 μg mL^−1^) than the crude extract, which exhibited a promising trypanocidal activity with a mild selectivity index (IC_50_ = 0.53 µg mL^−1^; selectivity index (SI) = 11.6) [21]. In fact, the enhanced activities of crude (thus more complex) extracts may be due to synergism effects, highlighting their potential without laborious and expensive fractionation/purification steps, often involving hazardous solvents. The exploitation of the trypanocidal activity of *B. bifurcata* extracts enriched in linear diterpenes may be important considering the gaps associated with sleeping sickness (trypanosomiasis) therapy, namely the fact that available drugs are outdated, causing severe adverse reactions [21].

Leishmaniosis, which is a common disease worldwide, affecting several mammal species, including humans [52], has been also the focus of different studies concerning the bioprospection of linear diterpenes from *B. bifurcata*. Antiprotozoal activity of EtOAc:MeOH soluble extract against *Leishmania donovani* (IC_50_ = 6.4 μg mL^−1^) was also shown [61]. Although, the EtOAc (10% *w*/*v*) extract was shown to be the most active against amastigotes of this strain, presenting an IC_50_ of 3.8 μg mL^−1^ [35]. Vonthron-Sé et al. also tested this active extract against erythrocytes infected by a resistant K1 strain of *Plasmodium falciparum* (100% of growth inhibition (GI) at 9.7 μg mL^−1^) and *Trypanosoma cruzi* trypamastigostes (78% of GI at 9.7 μg mL^−1^). These activities seem to be induced by toxicity [35]. Antiprotozoal activity of a *B. bifurcata* extract against *Trypanosoma brucei rhodeisense* (IC_50_ = 1.9 μg mL^−1^), *Trypanosoma cruzi* (IC_50_ = 34.7 μg mL^−1^), and *Mycobacterium tuberculosis* (MIC = 64.0 μg mL^−1^) (antitubercular activity) was also reported [61].

### 4.2. Antifouling Activity

Antifouling activity has been also proven in *B. bifurcata* extracts with high contents of linear diterpenes. The exploitation of this capacity of *B bifurcata* diterpenes could be an environmentally safe alternative for modern marine engineering and shipping operations, for offshore structures, and for aquaculture equipment [23,36]. In fact, the control of biofouling is an important problem of marine technology [23], with the use of heavy metal-based paints being already restricted due to the toxicity of organotin compounds to marine organisms [23,36]. Bacteria are commonly responsible for biofouling, forming a biofilm with other micro- and macrofoulers, such as algae or invertebrates, adhering to surfaces [14]. 

The antifouling activity of an Et_2_O *B. bifurcata* extract, in which two linear diterpenes were identified, was also tested against *Balanus amphitrite* cyprid, and was shown to be highly active (efficient concentration of extract to decrease microbial concentration by 50%, EC_50_ = 0.43 (0.88 − 1.26, conf. lim. 95%) μg mL^−1^), whereas eleganolone (**15**) (EC_50_ = 2.14 (0.88 − 3.9, conf. lim. 95%) μg mL^−1^) and eleganediol (**9**) (EC_50_ = 40.37 (11.89 – 62.93, conf. lim. 95%) μg mL^−1^) were shown to be moderate and low active, respectively. This study also proved that *B. bifurcata* extracts are toxic against *Balanus amphitrite* nauplius II after 24 h of treatment (lethal concentration (LC) = 0.64 (0.26 − 1.14, conf. lim. 95%) μg mL^−1^)). Compounds **9** (LC = 7.31 (3.84 − 8.78, conf. lim. 95%) μg mL^−1^) and **15** (LC = 3.48 (2.53 − 4.52, conf. lim. 95%) μg mL^−1^) showed lower toxicity [26]. The antiadhesion activity of compounds **9** and **15** were also studied against a strain of *Polibacter* sp. (EC_50_ = 63.5 ± 8.3 μg mL^−1^; EC_50_ = 58.4 ± 1.7 μg mL^−1^) and of *Paracoccus sp*. (EC_50_ = 103.9 ± 12.8 μg mL^−1^; EC_50_ = 40.3 ± 13.5 μg mL^−1^), with the component **15** being more active toward these two strains [25,44].

The antifouling activity of *B. bifurcata* linear diterpenes, considering the biofouling promoted by macroalgae and blue mussel, were also evaluated. The antifouling activity of eleganediol (**9**) was tested by Hellio et al., who proved that this linear diterpene is able to inhibit macroalgal spore and zygote development, particularly of *Enteromorpha intestinalis* (65.2% ± 1.2% GI) and *Sargassum muticum* (81.5% ± 3.4% GI), and to inhibit diatom growth (*Amphora coffeaformis* (37.3% ± 1.9% GI), *Phaeodartylum tricornutum* (42.8% ± 1.5% GI), and *Cylindrotheca closterium* (39.5% ± 1.6% GI)) [23]. Other linear diterpenes from *B. bifurcata*, such as 12-(*S*)-hydroxygeranylgeraniol (**1**) and geranylgeraniol (**39**) also showed antifouling activity toward macroalgal spores and zygotes. Compound **1** was shown to inhibit the development of *Sargassum muticum* (72.0% ± 2.2% GI) spore and zygote, whereas compound **39** was active against *Enteromorpha intestinalis* (67.3% ± 2.1% GI) and *Ulva lactuca* (83.1% ± 3.4% GI), as well as inhibiting the blue mussel *Mytilus edulis* adhesion (77.0% ± 2.8% inhibition of phenoloxidase activity (IPA)) [23].

Maréchal et al. studied the seasonal variation on the antifouling activity of crude *B. bifurcata* Et_2_O extracts (from Brittany, France) against cyprids of *Balanus amphitrite* and the marine bacteria *Cobetia marina* (MIC: 12.2 ± 0.4 to 26.3 ± 1.3 μg mL^−1^) and *Pseudoalteromonas haloplanktis* (MIC: 6.5 ± 0.6 to 15.5 ± 0.8 μg mL^−1^) [36].

Furthermore, the antifouling activity of ether extracts of *B. bifurcata* from different sampling sites was also tested. An extract of *B. bifurcata* from Quiberon, France was found to be active against *Ulva lactuca* spore and zygote development (78.7% ± 2.8% GI), whereas the Port Sall (France) and Oualidia (Morocco) *B. bifurcata* extracts inhibit the growth of diatoms (*Amphora coffeaformis* (83.9% ± 4.2% GI), *Phaeodartylum tricornutum* (86.2% ± 3.4% GI), *Cylindrotheca closterium* (69.2% ± 2.4% GI)) and inhibit the adhesion of the blue mussel *Mytilus edulis* (80.6% ± 2.3% IPA), respectively [23].

### 4.3. Antiproliferative Activity 

The cytotoxic activity of two bifurcadiol derivatives ((2*E*,10*E*,12*R*)-3,11,15-trimethyl-7-methylenehexadeca-2,10,14-triene-1,6,12-triol (**6**), and (2*E*,10*E*,12*R*)-3,7,11,15-tetramethylhexadeca-2,10,14-triene-1,7,12-triol (**7**) isolated from *B. bifurcata* were evaluated. These linear diterpenes were shown to be active against the NSCLC-N6 cell line (a human non–small-cell bronchopulmonary carcinoma line), presenting IC_50_ values of 12.3 and 9.5 μg mL^−1^, respectively) [44]. Eleganediol (**9**) and bifurcane (**10**), isolated from a *B. bifurcata* extract, showed anti-proliferative activity at 100 μg mL^−1^, toward MDA-MB-231 tumor cells (mammary gland/breast adenocarcinoma), resulting in 1.8% and 2.9% of cell viability, respectively [48].

Some studies also evaluated the in vitro antiproliferative activity of *B. bifurcata* extracts. Zubia et al. tested the cytotoxic activity of DCM:MeOH extracts of this brown macroalga against three tumoral cell lines (Daudi, Jurkat and K562), which resulted in, approximately, 40% of cell viability reduction with Daudi and K562 and about 20% of cell viability reduction with Jurkat cell lines [8]. Two carcinoma models (Caco-2 and HepG-2) were used to evaluate the in vitro antitumor activity of *B. bifurcata* extracts. In this research, Alves et al. showed high cell proliferation inhibition of both DCM and MeOH extracts (at 1 mg mL^−1^) against these tumoral cell lines. The IC_50_ values obtained with MeOH extract was 437.1 (266.0 – 718.1) μg mL^−1^ for Caco-2 and 252.0 (162.0 − 392.2) μg mL^−1^ for HepG-2. Although, DCM extract exhibited promising cell proliferation inhibition and cytotoxicity, against Caco-2 (IC_50_ = 82.31 (54.7 − 123.8) μg mL^−1^; IC_50_ = 90.09 (70.82 − 114.6) μg mL^−1^) and HepG-2 (IC_50_ = 95.63 (69.66 − 131.3) μg mL^−1^; IC_50_ = 123.9 (95.47 − 160.8) μg mL^−1^), when compared with the results obtained for cisplatin and tamoxifen, which are two commercial drugs [55].

In addition, Moreau et al. showed irreversible arrest of well-differentiated pathologic cells (such as NSCLC-N6) proliferation induced by a *B. bifurcata* MeOH extract rich in diterpenes. This antiproliferative effect is a mechanism of action that overcomes the limited effectiveness of many cycle-dependent anticancer drugs on such slowly developing tumors. After 72 h of treatment with *B. bifurcata* extract (2.5 μg L hours^−1^), the cell growth in the G1 phase of the cell cycle was inhibited, and kinetic assays in pretreated cells proved that this growth arrest was irreversible [37].

Besides these results regarding anti-proliferative activity of *B. bifurcata* linear diterpenes and extracts, bioaccessibility and bioavailability assays were not yet performed. In fact, most of the in vitro studies have not used gut cell lines, which allow consideration of the absorption and distribution stages. Thus, other in vitro assays are required in the future to complement these results.

### 4.4. Antioxidant Activity

The prospection of natural components from macroalgae with antioxidant activity has been object of several studies, since oxidative stress is known to be related with several diseases, such as cancer, chronic inflammation, atherosclerosis, and cardiovascular disorder, among others [8]. Furthermore, consumers have, currently, preference for products from natural sources and are conscious about the toxicity associated with synthetic antioxidants [8]. The industrial implementation of natural antioxidants, whether at the level of food industry or the cosmetic or therapeutic industry (i.e., nutraceuticals), appears as a promising alternative to synthetic antioxidants [54]. This may be one of the reasons why antioxidant activity has been one of the most studied biological activities in *B. bifurcata* extracts [8,38,54,55]. 

The antioxidant potential of *B. bifurcata* linear diterpenes and extracts has been evaluated by different assays, such as 2,2-diphenyl-1-picrylhydrazyl (DPPH), 2,2’-azino-bis(3-ethylbenzothiazoline-6-sulphonic acid (ABTS), and oxygen radical absorbent capacity (ORAC). The results of DPPH and ABTS assays are commonly expressed as IC_50_ values, defined as the inhibitory concentration of the extract required to decrease by 50% the initial radical concentration. Nonetheless, the comparison of the different IC_50_ values between published studies is not always possible, since the IC_50_ value depends on the methodology used by each author, as well as on the standards used. For example, Santos et al. used ascorbic acid and 3,5-di-tert-4-butylhydroxytoluene (BHT) as positive control whereas Pinteus et al. used only BHT [38,54]. Despite earlier studies, namely from Santos et al. and Alves et al., using the same standards as positive control, the DPPH assay conditions were different, which make the results incomparable [38,55].

Zubia et al. determined the antioxidant activity of *B. bifurcata* crude DCM:MeOH extract by three methods: DPPH (efficient concentration of extract to decrease reagent concentration by 50%, EC_50_ = 0.56 ± 0.00 mg mL^−1^), reducing activity (90.97% at 500 mg L^−1^), and *β*-carotene-linoleic acid system (76.13% of inhibition at 500 mg L^−1^) [8]. A DCM extract, obtained from a *B. bifurcata* sample of Ria de Aveiro, Portugal, was evaluated by DPPH and ABTS assays, exhibiting IC_50_ values of 365.57 ± 10.04 μg mL^−^^1^ and 116.25 ± 2.54 μg mL^−^^1^ for DPPH and ABTS, respectively [38]. Alves et al. also carried out DPPH assay in both MeOH (IC_50_ = 58.82 (50.65 − 68.31) μg mL^−^^1^) and DCM (IC_50_ = 344.70 (246.10 − 482.80) μg mL^−^^1^) extracts of a *B. bifurcata* from Peniche Coast, Portugal, however with MeOH extract showing a higher antioxidant activity than DCM extract [54,55]. Notwithstanding, the DCM extract showed an IC_50_ value close to that obtained in a similar study [38,54,55]. In addition to DPPH assay, the ORAC of MeOH (3151.35 ± 119.33 μmol trolox equivalents (TE) g^−1^ extract (ext)) and DCM (589.98 ± 7.33 μmol TE g^−1^ ext) extracts were determined [54,55].

### 4.5. Anti-Inflammatory Activity

The anti-inflammatory activity of a *B. bifurcata* extract enriched in linear diterpenes was described by Santos et al., who analyzed a DCM extract from a Portuguese sample [38]. The capacity of this extract to modulate nitric oxide (NO) was evaluated through an in vitro model of inflammation consisting of macrophages stimulated with lipopolysaccharides (LPS) and, simultaneously, the cell viability was analyzed by the resazurin-based assay, which allowed researchers to select concentrations with bioactivity and without cytotoxicity. The accumulation of nitrites in the culture medium, using the Griess assay, was measured to determine the extract effect on NO production. Thus, LPS-induced NO production was 6% inhibited in the presence of the extract at a concentration of about 50 μg mL^−^^1^ [38].

### 4.6. Other Biological Activities

Two components widely reported in *B. bifurcata* samples, namely eleganediol (**9**) and eleganolone (**15**), were studied regarding antihypertensive and relaxing activities. These components were isolated from *Cystoseira balearica*. These pharmacological assays were performed on different guinea pig intestinal preparations, and allowed researchers to identify bioactive effects of compounds **9** and **15**, such as blocking the isoprenaline inotropic activity and inhibiting the contractile activities of acetylcholine (pIC_50_ = 4.71 ± 0.18 μg mL^−1^; pIC_50_ = 4.60 ± 0.03 μg mL^−1^, respectively) and histamine (pIC_50_ = 5.18 ± 0.01 μg mL^−1^; pIC_50_ = 4.84 ± 0.20 μg mL^−1^, respectively) on ileum musculature. In addition, compounds **9** and **15** relaxed, in a dose-dependent manner, the same preparations precontracted with 300 mM BaCl_2_ (pIC_50_ = 4.34 ± 0.02 μg mL^−1^; pIC_50_ = 4.34 ± 0.02 μg mL^−1^, respectively) or with 600 mM KCl (pIC_50_ = 4.47 ± 0.06 μg mL^−1^; pIC_50_ = 4.73 ± 0.18 μg mL^−1^, respectively) [53,64].

Silva et al. proved that a *B. bifurcata* DCM extract also presents neuroprotective potential. Neuroprotective effects were highlighted in a neurotoxic model induced in a human neuroblastoma cell line (SH-SY5Y), whereas the neuroprotection mechanisms were evaluated by the determination of mitochondrial membrane potential, H_2_O_2_ production, among others. After fractionation, the cyclohexane-EtOAc (1:2) fraction was shown to have a promising neuroprotective performance, due to its ability to prevent changes in mitochondrial potential (218.10% ± 14.87% of control), and to induce the reduction of H_2_O_2_ levels of production (204.50% ± 15.12% of control), as well as to revert neurotoxic effect on cell viability (about 20%–25%). Considering these results, Silva et al. investigated the composition of this fraction, through purification steps, in order to isolate the potential bioactive molecules. Eleganolone (**15**) and eleganolal (**23**) were the two major compounds of this fraction. Then, their antioxidant activity was evaluated, demonstrating lower ability to reduce the DPPH radical, when compared to the fraction from which they were isolated. Although, taking into account the results of FRAP and ORAC assays, compounds **15** (8341.18 ± 177.72 µM FeSO_4_ g^−1^ compound and 1663.83 ± 25.35 µmol TE g^−1^ compound, respectively) and **23** (8635.37 ± 389.54 µM FeSO_4_ g^−1^ compound and 667.48 ± 10.96 µmol TE g^−1^ compound, respectively) expressed antioxidant capacity, since these linear diterpenes exhibited a high potential in reducing peroxyl radicals and have a strong iron reduction capacity, compared to the BHT. In this sense, these components might be promising candidates for further neuroprotection studies [51].

### 4.7. In Vitro Estimation of Toxicity

The evaluation of biological dose effects should be combined with the respective assessment of toxicity, since this is a crucial parameter to ensure that bioactive compounds and/or extracts are safe for humans.

The cytotoxicity of linear diterpenes obtained from a *B. bifurcata* sample, collected in Brittany, France, was evaluated using sea urchin eggs, which have been considered a model for studies on cell division and embryologic development and commonly used to obtain a general screening of cytotoxicity [14]. The ability of some linear diterpenes to inhibit the development of fertilized eggs of the common sea urchin *Paracentrotus lividus* was evaluated through a cytotoxicity activity test [29,43]. The test showed that 12-(*S*)-hydroxygeranylgeraniol (**1**), bifurcane (**10**), and bifurcanol (**41**) were active, with EC_50_ values of 18 μg mL^−^^1^, 12 μg mL^−^^1^, and 4 μg mL^−^^1^, respectively. Whereas, (*S*)-12-hydroxygeranylgeranic acid (**2**) and eleganediol (**9**) exhibited moderate antimitotic activity with EC_50_ values of 60 μg mL^−^^1^ and 36 μg mL^−^^1^, respectively [29,43].

The cytotoxicity of nine linear diterpenes isolated from *B. bifurcata* were tested by Göthel et al. [30]. Bifurcane (**10**), bifurcanone (**18**), eleganonic acid (**31**), and bibiolone (**34**) were shown to be inactive (when IC_50_ > 40 µg mL^−1^) against mouse fibroblast cell line (L929), whereas eleganolone (**15**) (IC_50_ = 22 μg mL^−1^), (6*E*,10*E*,14*E*)-16-hydroxy-2,6,10,14-tetramethylhexadeca-2,6,10,14-tetraen-4-one (**21**) (IC_50_ = 18 μg mL^−1^), eleganolonebutenolide (**32**) (IC_50_ = 27 μg mL^−1^), 14,15-dihydro-eleganonic acid (**33**) (IC_50_ = 20 μg mL^−1^), and bifurcanol (**41**) (IC_50_ = 24 μg mL^−1^) exhibited very low toxicity [30]. In the case of compound **15**, for example, the cytotoxicity IC_50_ value was higher than the EC_50_ found for antifouling activity (against *Trypanosoma brucei rhodesiense* (IC_50_ = 13.7 µg mL^−1^), *Trypanosoma cruzi* (IC_50_ = 17.7 µg mL^−1^), *Plasmodium falciparum* (IC_50_ = 2.6 µg mL^−1^), and *Balanus amphitrite cyprid* (EC_50_ = 2.14 (0.88−3.9, conf. lim. 95%) μg mL^−1^) [21,26]. 

For instance, Smyrniotopoulos et al. [50] analyzed the cytotoxicity of bifurcatriol (**14**), showing that this linear diterpene had cytotoxicity against the L6 rat myoblast cell line, IC_50_ = 56.6 ± 0.004 µg mL^−1^ [50]. Actually, this value is considerably higher that the IC_50_ values observed for antimalarial and antiprotozoal activities, compromising, therefore, the use of this compound for such purposes. 

In fact, other studies have shown biological activities and cytotoxicity of *B. bifurcata* extracts, with both expressed at µg mL^−1^ [35,61]. Notwithstanding, these toxicities corresponded to EtOAc and EtOAc:MeOH extracts, with the toxicity of the most relevant and studied extracts, namely DCM and EtOH extracts, remaining unknown. In addition, considering bioactive extracts in which a high toxicity is present, the hypothesis that bioactive and toxic components are not the same must be carefully taken into account. Therefore, it is crucial to investigate the compounds responsible for the biological activities found, as well as evaluating their toxic nature. Thus, strategies to separate the bioactive compounds from those with high toxicity must be developed. In vitro cytotoxicity studies must be accomplished for interspecies correlations. Hence, to ponder a potential application of a bioactive compound and/or extract, it is essential to ensure that the magnitude order of bioactivity is significantly higher than those of toxicity.

In vitro toxicity assays involving marine bacteria should also be considered in the future, since equations for interspecies dose correlations have already been established, allowing researchers to correlate the bacteria data with rat or mouse toxicity, which avoids mammalian laboratory tests [65,66].

Finally, the safe use of these components must be verified by additional studies, in particular in vivo assays, considering the different range of possible applications, in order to confirm that they are safe to humans.

## 5. Potential Applications for *B. bifurcata* Extracts

Taking into account the diverse biological activities already attributed to *B. bifurcata* linear diterpenes, different studies have already exploited specific applications for these components or enriched extracts. Miranda et al. studied the effect of a preliminary dipping treatment containing a *B. bifurcata* extract in the fish quality during chilled storage. The choice of this brown macroalga was due to its content on antioxidant and antimicrobial compounds, i.e., diterpenes, among others [67]. Through microbial analysis, the inhibitory effects against Enterobacteriaceae, lipolytic, and psychotropic bacteria were proven. These results can be explained by two combined effects: the removal through the washing effect of blood, digestive juices, slime, and feces from the fish surface; and the presence of a high content of antimicrobial diterpenes from the algae extract. Chemical assays, in particular, trimethylamine and free fatty acids formation, allowed researchers to prove the enhancement of fish quality in the presence of algae extracts. Although, these effects were found in specimens submitted to the most concentrated algae extract, at advanced storage time (in most cases), which means that the combined treatment, including washing in an ethanolic-aqueous solution and the inclusion of a bioactive *B. bifurcata* extract, had beneficial effects on fish quality. In fact, the quality during chilled storage is a major concern of the food industry. Wild marine species are often exposed to several handling and technological processes, which determine the quality of the final product [67]. A water dipping step is often applied before storage to remove blood, slime, and other undesirable components and to partially prevent microbial contamination. This preliminary step is also carried out to avoid the development of different damage pathways, resulting from microbial activity, autolysis and lipid oxidation, among others [67]. Thus, the inclusion of a bioactive *B. bifurcata* extract may be a practical application for both on-board and in-land fish storage strategies [67]. 

The use of a *B. bifurcata* extract in the green synthesis of copper oxide nanoparticles (CONPs) was also reported. This strategy aimed to overcome some drawbacks of conventional processes (such as the use of toxic chemicals as reducing and capping agents of nanoparticles), which limit the use of nanoparticles in applications related to clinical fields. The synthesis of CONPs using this marine brown macroalga was focused on “green” chemistry and bioprocess approaches [68]. In addition to being an environmental friendly bioprocess (no chemical reagents or surfactant templates were applied), these CONPs showed good antibacterial activity against gram-negative (*Enterobacter aerogenes*) (ZI = 14 mm) and gram-positive (*Staphylococcus aureus*) (ZI = 16 mm) bacteria [68].

## 6. Conclusions and Future Perspectives

Macroalgae are one of the most promising sources of secondary metabolites, which have already been associated to several biological activities. For this reason, an increase in the investment and research on these marine resources has been observed. The focus of this review was the bioprospection of linear diterpenes from *B. bifurcata,* including the methodologies of extraction and structural elucidation and the biological activities already associated to these brown macroalga components. A detailed collection of NMR and MS data is provided, which will be of utmost importance in future studies of bioprospection of linear diterpenes from *B. bifurcata*.

*B. bifurcata* has earned special emphasis, due to their special ability to biosynthesize linear diterpenes, which are rarely found in brown macroalgae species outside the Sargassaceae family. In fact, some studies have proven that these secondary metabolites are responsible for several biological activities, such as antibacterial, antiprotozoal, antifouling, and antitumoral, among others. Linear diterpenes from *B. bifurcata*, particularly eleganolone (**15**) and eleganediol (**9**), showed significant antifouling, antibacterial, and antihypertensive activities in the range of µg mL^−1^.

Notwithstanding, extraction methodologies have been identified as one of the major challenges to develop viable and safe high-value applications, namely in food, nutraceutical, or pharmaceutical fields. The conventional extraction methodologies normally used to extract these compounds involve the use of organic solvents, often toxic to humans and environmentally hazardous. Furthermore, these extraction methodologies also raise other concerns, namely, the long operation times, consumption of high volumes of expensive solvents, and high energetic demand. Thus, new and eco-friendly methodologies should be applied, taking into account their overall feasibility. In particular, emergent extraction methodologies able to use small amounts of organic solvents, or even offering the possibility for their replacement by water, should be considered. 

The purification and/or fractionation steps have been already applied by some authors, frequently for identification purposes, for *B. bifurcata*, although there are also studies reporting biological activities of *B. bifurcata* extracts without isolating the bioactive compounds. The isolation, or at least the fractionation, should be considered in order to enhance the bioactivity and also eliminate some antagonistic effects, for instance, bio-guided fractionation or a faster strategy that has already been tested, known as “pharmacophoric deconvolution”, should be further exploited. Another strategy could be the development and optimization of more selective extraction methodologies. Additionally, before any possible application, the cytotoxicity of the extracts, fractions, or isolated compounds should be evaluated. 

In vivo assays for the determination of dose effect and the consumer security evaluation may be also considered to confirm the potential of *B. bifurcata* linear diterpenes in pharmaceutical, cosmetic, or nutraceutical applications. In addition, the incorporation of these extracts in biomaterials should be considered. Finally, for possible food industry applications, the legislation may be revised in order to evaluate *B. bifurcata* as an edible species.

Finally, and despite the use of metabolic engineering and synthetic biology in macroalgae’s bioactive components, production is still in its infancy and largely unexploited in *B. bifurcata*, although the new advances in these fields should be undoubtably considered in future works. Actually, the increasing development of multi-omics techniques, including genomics, transcriptomics, proteomics, and metabolomics have enabled the creation of libraries and models that can predict the behavior of biological systems as well as allow the optimization of cellular processes in order to produce target compounds. This could be a valuable and key strategy in the exploitation of *B. bifurcata* as a source of linear diterpenes for high-value applications. 

## Figures and Tables

**Table 1:** Linear diterpenes extracted from *B. bifurcata*, and the respective pretreatment, extraction, purification/fractionation and identification/analysis methodologies used, and time of collection/geographical origin.

Extraction Methodology	EY ^a^	Sample Pretreatment	Fractionation/Purification	Identification/Analysis	Compound (Content ^b^)	Time of Collection/Geographical. Origin	Ref
EtOAc S–L extraction	1.70	Freeze-dried	Chromatography (on Silica gel) eluted with EtOAc-*n*-heptane (4:1) and monitored at 270 nm; Bio-guided fractionation: chromatography (on a Lobar RP-8 column) with EtOH-H_2_O; Fr. purification with MeCN-H_2_O or EtOAc-CHCl_3_.	(^1^H and ^13^C) NMR, MS	**9** (0.06)	July to August/Roscoff, Brittany, France	[28]
					**15** (0.07)		
					**18** (0.11)		
					**19** (0.03)		
					**20** (0.02)		
EtOAc S–L extraction	N.D.	Freeze-dried	LC using Silica gel with a solvent gradient from DCM to DCM-MeOH (80:20); CC (on Silica gel) with solvent gradient DCM-MeOH (100:0 to 97:3) HPLC (RP18 column) with a gradient elution (from H_2_O-MeCN (60:40), H_2_O-MeCN (80:20) to MeOH)	HRESIMS, 1D and 2D NMR	**10**	September/Roscoff, Brittany, France	[30]
					**15**		
					**18**		
					**21**		
					**27**		
					**31**		
					**32**		
					**33**		
					**34**		
					**41**		
EtOAc S–L extraction, at RT	4.80	Freeze-dried and ground	Successive flash and CC (on Silica gel), eluting with cyclohexane—EtOAc mixture; Bio-guided fractionation: CC (on Silica gel), with cyclohexane-EtOAc	HREIMS, (^1^H and ^13^C) NMR	**15** (0.004)	November to September/Basse-Normandie, France	[21]
EtOAc S–L extraction, at RT	N.D.	Freeze-dried and ground	Flash chromatography eluting with a H_2_O-MeOH mixture of increasing polarity (95:5–0:100 in 30 minutes)	2D NMR, HPLC-DAD-MS-SPE-NMR	**15**	June/Cap Lévi, English Channel, France	[22]
EtOAc S–L extraction, at RT	4.8	Freeze-dried and ground	CC (on Silica gel) eluted with a solvent gradient from DCM to DCM-MeOH (80:20) UV-ESI-DAD-HPLC (using a Kromasil RP18 column) eluted with a solvent gradient H_2_O-MeCN-MeOH	(^1^H and ^13^C) NMR, HRESI(+)MS	**35** (0.002)	September/Roscoff, Brittany, France	[34]
					**36** (0.001)		
					**44** (0.0004)		
CHCl_3_ S–L extraction	N.D.		Normal and reverse phase flash CC and RP18 HPLC	1D and 2D NMR, HRMS	**9**	May/Kilkee, County Clare of Ireland	[48]
					**10**		
CHCl_3_-EtOH S–L extraction	1.52	Freeze-dried and ground	Partitioning between H_2_O and Et_2_O; Et_2_O-soluble material: CC (on Silica gel) eluted with hexane-EtOAc (2:3); HPLC (EtOAc–isooctane, 2:3), with RI monitoring	IR, (^1^H and ^13^C) NMR, EIMS	**1** (0.86)	-/Atlantic coast Morocco	[45]
CHCl_3_-MeOH S–L extraction, at RT	5.53	Shade-dried and ground	Partitioning in the mixture MeOH-isooctane (1:1); MeOH phase: dissolved in the mixture MeOH-CHCl_3_-H_2_O (4:3:1); Organic phase: CC (on Silica gel) eluted with a solvent gradient from isooctane to EtOAc and then from EtOAc to MeOH.	HREIMS, (^1^H and ^13^C) NMR	**1** (0.37)	December/Oualidia, Morocco	[44]
					**6** (0.01)		
					**7** (0.004)		
CHCl_3_- MeOH S–L extraction; Et_2_O S–L extraction of aqueous phase	1.95 g ext	Freshly collected	Open CC (on Silica gel) eluted with hexane to EtOAc (2:3); HPLC (eluent EtOAc-isooctane, 2:3).	IR, UV, (^1^H and ^13^C) NMR, EIMS, HRMS	**2** (0.17)	-/Morocco	[47]
					**40** (0.02)		
CHCl_3_-MeOH S–L extraction	2.63	Shade-dried	Partitioning in the mixture MeOH-isooctane (1:1); MeOH extract: dissolution in the mixture MeOH-CHCl_3_-H_2_O (4:3:1); Organic extract: fractionation on silica gel column eluted with EtOAc and EtOAc-MeOH (98:2 and 95:5); HPLC on C-18 reversed-phase column, eluting with MeCN-H_2_O (1:1 and/or 2:3).	HRMS, IR, (^1^H and ^13^C) NMR	**8**	-/Oualidia, Morocco	[49]
					**43**		
CHCl_3_-MeOH S–L extraction, at RT CHCl_3_-H_2_O L–L extraction	6.92	Air-dried and ground	MeOH extract: defatting with *n*-hexane; CC (on Silica gel) using mixtures of CHCl_3_-MeOH (from CHCl_3_ to CHCl_3_-MeOH, 1:1).	HPLC	**9**	July/Quiberon, Brittany, France	[37]
					**13**		
					**15**		
					**21**		
					**25**		
					**26**		
					**27**		
					**28**		
					**29**		
					**30**		
					**39**		
CHCl_3_- MeOH S–L extraction, at RT	3.11	Shade-dried and ground	CC (on Silica gel) eluted with a solvent gradient from cyclohexane to EtOAc and then from EtOAc to MeOH; HPLC on an analytical C-18 reverse-phase column (eluent, MeCN-H_2_O), with RI monitoring	HREIMS, IR, (^1^H and ^13^C) NMR	**9** (0.15)	July/Quiberon, Brittany, France	[24]
					**13** (0.0001)		
					**15** (0.40)		
					**21** (0.01)		
					**25** (0.0001)		
					**26** (0.0003)		
					**27** (0.0002)		
					**28** (0.0001)		
					**29** (0.0005)		
					**30** (0.0005)		
					**39** (0.008)		
					**47** (0.01)		
Et_2_O S–L extraction, at RT	1.65	Freeze-dried and powder	Partitioning between H_2_O and Et_2_O; Et_2_O-soluble material: CC (on Silica gel) eluted with a solvent gradient from hexane to EtOAc; HPLC (eluent, EtOAc–isooctane), with RI monitoring	HRMS, EIMS, IR, (^1^H and ^13^C) NMR	**1** (0.33–0.34)	January to December/Atlantic coast of Morocco	[43]
					**2** (0.07–0.10)		
					**9** (0.10–0.12; 0.36–0.38)		
					**15** (0.29–0.32)		
					**40** (0.03)		
					**41** (0.01;0.04–0.05)		
Et_2_O S–L extraction	2.94	Air-dried and ground	CC (on Silica gel) eluted with a gradient from isooctane to EtOAc; HPLC (eluent, isooctane-EtOAc); Semi-preparative and then analytical normal-phase HPLC with RI-monitoring.	1D and 2D NMR	**1** (0.71)	November/Oualidia, Morocco	[23]
					**39** (0.12)		
	3.15				**9** (0.14)	December/Quiberon, Brittany, France	
					**15** (0.47)		
					**21** (0.01)		
					**22** (0.01)		
					**25** (0.02)		
Et_2_O S–L extraction, at RT	2.94	Shade-dried and ground	CC (on Silica gel) eluted with a solvent gradient from isooctane to EtOAc Semi-preparative normal phase HPLC (eluent, EtOAc-isooctane), with RI monitoring	HRMS, (^1^H and ^13^C) NMR	**1** (0.71)	December/Oualidia, Morocco	[46]
					**2** (0.03)		
					**3** (0.08)		
					**4** (0.01)		
					**5** (0.004)		
					**39** (0.12)		
					**42** (0.01)		
Et_2_O S–L extraction	N.D.	Freeze-dried	Preparative HPLC (DCM-EtOAc, 90:10)	UV, IR, (^1^H and ^13^C) NMR, MS	**9** (0.28)	-/Loire-Atlantique, France	[19]
					**16** (0.06)		
Et_2_O S–L extraction	N.D.	Shade-dried	Separation in different fractions		**9**	-/Brittany, France	[26]
					**15**		
Et_2_O S–L extraction, at RT, for 48h	2.2-2.9	Air-dried and ground	CC (on Silica gel) eluted with a solvent gradient from isooctane to EtOAc; Normal-phase HPLC (eluent, isooctane-EtOAc, 2:3), with RI-monitoring	IR, UV, HRMS, 1D and 2D NMR	**9** (1.12)	July to June/Roscoff, Brittany, France	[36]
					**10** (0.37)		
					**15** (0.06)		
					**39** (0.02)		
Et_2_O S–L extraction, at RT	2.40	Freeze-dried and ground	Partitioning between H_2_O and Et_2_O; Et_2_O-soluble material: CC (on Silica gel) eluted with hexane-EtOAc (1:1); HPLC (EtOAc–isooctane), with RI monitoring.	HRMS, EIMS, IR, (^1^H and ^13^C) NMR	**9** (0.15)	July to August/Roscoff, Brittany, France	[29]
					**10** (0.23)		
					**11** (0.03)		
Et_2_O S–L extraction, at RT	3.15	Shade-dried and ground	CC (on Silica gel) eluted with a solvent gradient from isooctane to EtOAc; Semi-preparative normal-phase HPLC (eluent, EtOAc-isooctane), with RI monitoring	HRMS, EIMS, IR, (^1^H and ^13^C) NMR.	**9** (0.06)	December/Quiberon and Roscoff, Brittany, France	[31]
					**15** (0.01)		
					**21** (0.01)		
					**22** (0.02)		
					**25** (0.03)		
	2.40		CC (on Silica gel) eluted with a solvent gradient from hexane to Et_2_O; HPLC (EtOAc- isooctane, 2:3), with RI monitoring.		**9** (0.04)		
					**10** (0.06)		
					**11** (0.01)		
					**12** (0.007)		
					**15** (0.003)		
Et_2_O S–L extraction	N.D.	Dried	LC using Silica gel; HPLC of the most polar fraction;	IR, MS, NMR	**9**	-/Quiberon, Brittany, France	[33]
					**25**		
					**47**		
Et_2_O S–L extraction, at RT	N.D.	Crushed and freeze-dried	Insoluble impurities and pigments elimination with isooctane and EtOH-H_2_O, respectively; TLC (eluted with Et_2_O-petroleum ether, 50:50 or DCM-EtOAc 70:30); CC (on Silica gel, eluted with Et_2_O-petroleum ether, 75:25) Semi-preparative HPLC	UV, IR, (^1^H and ^13^C) NMR, MS	**15** (2)	-/Loire-Atlantique, France	[20]
Et_2_O S–L extraction (3x) (3h at 20ºC)	3.67	Freeze-dried and powder	CC (on Silica gel) eluted with a solvent gradient from hexane to Et_2_O More polar fr.: HPLC with DCM-EtOAc (90:10)	IR, UV, MS, (^1^H and ^13^C) NMR	**15**	January to December/Piriac, France	[27]
					**17** (0.01)		
Et_2_O S–L extraction	N.D.	Dried	LC using Silica gel; HPLC of the less polar fr.; Semi-preparative normal-phase HPLC (EtOAc-isooctane, 1:1).	IR, MS, NMR	**23** (0.12)	-/El Jadida, Morocco	[32]
					**24** (0.08)		
					**39** (0.01)		
DCM S–L extraction, at RT; MeOH S–L extraction	9.06	Freeze-dried	Modified Kupchan method: partitioning between (90:10) MeOH-H_2_O and *n-*hexane; MeOH-H_2_O phase: partitioning between (65:35) MeOH-H_2_O and CHCl_3_; Fractionation by flash chromatography (on Silica gel), with EtOAc-*n*-hexane (80:20) DAD and ELSD-HPLC (using a reverse-phase column), eluent gradient (55:45) MeCN-H_2_O for 13 min, increasing to 100% MeCN in 5 min, maintaining for 20 min (rt = 16.3 min)	IR, 1D and 2D NMR, HRMS, VCD	**14** (0.007)	May/Kilkee, County Clare of Ireland	[50]
MeOH S–L extraction DCM S–L extraction	0.95	Freeze-dried	VLC (on Silica gel) eluted with cyclohexane-EtOAc (1:2); Semi-preparative reverse phase HPLC (gradient of H_2_O-MeCN) PTLC over Silica gel (*n*-hexane-EtOAc, 7:3)	(^1^H, ^13^C, APT, COSY, HMBC and HSQC) NMR	**15** (0.002)	May to June/Peniche, Portugal	[51]
					**23** (9.5 × 10^−5^)		
DCM Soxhlet extraction (9 h)	3.92	Freeze-dried and ground		GC-MS	**37** (0.06)	May/Ria de Aveiro, Portugal	[38]
					**38** (0.10)		
					**39** (0.01)		
					**42** (0.01)		
					**45** (0.003)		
					**46** (0.01)		

^a^—% (*w*/*w*); ^b^—when available, % of dw; ^1^H—proton; ^13^C—carbon; 1D and 2D—one and two dimensional; APT—attached proton test; CC—column chromatography; COSY—correlated spectroscopy; DAD—diode array detector; DCM—dichloromethane; EIMS—electron ionization mass spectrometry; ELSD—evaporative light scattering detector; ESI—electrospray ionization; Et_2_O—diethyl ether; EtOAc—ethyl acetate; EtOH—ethanol; EY—extraction yield; ext.—extract; fr.—fraction; HMBC—heteronuclear multiple bond correlation; HPLC—high performance liquid chromatography; HRMS—high resolution mass spectrometry; HSQC—heteronuclear single quantum coherence; IR—infrared spectroscopy; LC—liquid chromatography; L–L—liquid–liquid; MeCN—acetonitrile; MeOH—methanol; MS—mass spectrometry; N.D.—nondeterminate; NMR—nuclear magnetic resonance; PTLC—plate thin layer chromatography; Ref—reference; RI—refractive index detector; rt—retention time; RP—reverse phase; RT—room temperature; S–L—solid–liquid; SPE—solid phase extraction; TLC—thin-layer chromatography; UV—ultraviolet spectroscopy; VCD—vibrational circular dichroism; VLC—Vacuum liquid chromatography.

**Table 2:** *B. bifurcata* linear diterpenes and crude extracts’ biological activities.

Compounds/Crude Extract (Yield)	Biological Activities	Ref.
12-(*S*)-hydroxygeranylgeraniol (**1**)	Antimitotic activity (assay of cytotoxicity activity—inhibition of development of fertilized eggs of the common sea urchin *Paracentrotus lividus*—EC_50_ = 18 μg mL^−^^1^)	[43]
	Antifouling activity toward macroalgae spore and zygote development (*Sargassum muticum* (72.0% ± 2.2% GI))	[23]
(*S*)-12-hydroxygeranylgeranic acid (**2**)	Antimitotic activity (assay of cytotoxicity activity—inhibition of development of fertilized eggs of the common sea urchin *Paracentrotus lividus*—EC_50_ = 60 μg mL^−^^1^)	[43]
(2*E*,10*E*,12*R*)-3,11,15-trimethyl-7-methylenehexadeca-2,10,14-triene-1,6,12-triol (**6**)	Cytotoxic activity: inhibit in vitro proliferation of pathogenic cells (NSCLC-N6—derived from a human non–small-cell bronchopulmonary carcinoma) by terminal differentiation. IC_50_ = 12.3 μg mL^−^^1^	[44]
(2*E*,10*E*,12*R*)-3,7,11,15-tetramethylhexadeca-2,10,14-triene-1,7,12-triol (**7**)	Cytotoxic activity: inhibit in vitro proliferation of pathogenic cells (NSCLC-N6—derived from a human non–small-cell bronchopulmonary carcinoma) by terminal differentiation. IC_50_ = 9.5 μg mL^−^^1^	[44]
Eleganediol (**9**)	Antibacterial activity (against *Bacillus* sp. (MIC = 8 μg mL^−^^1^)	[23]
	Antifouling activity toward macroalgal spore and zygote development (*Enteromorpha intestinalis* (65.2 ± 1.2% GI), *Sargassum muticum* (81.5 ± 3.4% GI)), and against diatom growth (*Amphora coffeaformis* (37.3% ± 1.9% GI)*, Phaeodartylum tricornutum* (42.8% ± 1.5% GI)*, Cylindrotheca closterium* (39.5% ± 1.6% GI))	
	Antimitotic activity (assay of cytotoxicity activity—inhibition of development of fertilized eggs of the common sea urchin *Paracentrotus lividus*—EC_50_ = 36 μg mL^−^^1^)	[43]
	Antiadhesion activity (against a strain of *Polibacter* sp. (EC_50_ = 63.5 ± 8.3 μg mL^−^^1^) and of *Paracoccus* sp. (EC_50_ = 103.9 ± 12.8 μg mL^−^^1^)	[25]
	Antifouling activity (against *Balanus amphitrite* cyprid (EC_50_ = 40.37 (11.89 − 62.93, conf. lim. 95%) μg mL^−^^1^)	[26]
	Toxicity (against *Balanus amphitrite* nauplius (LC = 7.31 (3.84 − 8.78, conf. lim. 95%) μg mL^−^^1^))	
	Antiproliferative activity toward MDA-MB-231 tumor cells (1.8% cell viability at 100 μg mL^−^^1^)	[48]
Bifurcane (**10**)	Antimitotic activity (assay of cytotoxicity activity - inhibition of development of fertilized eggs of the common sea urchin *Paracentrotus lividus* – EC_50_ = 12 μg mL^−^^1^).	[29]
	Antiproliferative activity toward MDA-MB-231 tumor cells (2.9% cell viability at 100 μg mL^−^^1^)	[48]
Bifurcatriol (**14**)	Antimalarial activity (against resistant K1 strain of the malaria parasite, *Plasmodium falciparum* – IC_50_ = 0.65 ± 0.05 μg mL^−^^1^)	[50]
	Antiprotozoal activity: *Trypanosoma brucei rhodesiense* (IC_50_ = 11.8 ± 0.01 μg mL^−^^1^), *Trypanosoma cruzi* (IC_50_ = 47.8 ± 0.59 μg mL^−^^1^)*, Leishmania donovani* (IC_50_ = 18.8 ± 0.12 μg mL^−^^1^))	
	Cytotoxicity against the L6 rat myoblast cell line (IC_50_ = 56.6 ± 0.004 μg mL^−^^1^)	
Eleganolone (**15**)	Antimicrobial activity (against *Mycobacterium smegmatis* (75 μg mL^−^^1^), *Bacillus subtilis* (2.5 mg mL^−1^), *Mycobacterium aquae* (400 μg mL^−^^1^), *Mycobacterium ranae* (100 μg mL^−^^1^)*, Mycobacterium xenoqui* (200 μg mL^−^^1^)*, Mycobacterium avium* (100 μg mL^−^^1^))	[20]
	Antiprotozoal activity (against *Trypanosoma brucei rhodesiense* (IC_50_ = 13.7 μg mL^−^^1^), *Trypanosoma cruzi* (IC_50_ = 17.7 μg mL^−^^1^), *Plasmodium falciparum*, IC_50_ = 2.6 μg mL^−^^1^ (SI = 21.6))	[21]
	In vitro antiplasmodial activity (against *Plasmodium falciparum* 7G8 strain – antimalarial activity – In fraction: 47% of growth inhibition at 10 μg mL^−^^1^)	[22]
	Antibacterial activity (against *Bacillus* sp. (MIC = 8 μg mL^−^^1^)	[23]
	Anti-adhesion activity (against a strain of *Polibacter* sp. (EC_50_ = 58.4 ± 1.7 μg mL^−^^1^) and of *Paracoccus* sp. (EC_50_ = 40.3 ± 13.5 μg mL^−^^1^)	[25]
	Antifouling activity (against *Balanus amphitrite* cyprid (EC_50_ = 2.14 (0.88 − 3.9, conf. lim 95%) μg mL^−^^1^)	[26]
	Toxicity (against *Balanus amphitrite* nauplius (LC = 3.48 (2.53 − 4.52, conf. lim 95%) μg mL^−^^1^))	
	Cytotoxicity (against mouse fibroblast cell line (L929), IC_50_ = 22 μg mL^−^^1^)	[30]
	Antioxidant potential (ORAC—1663.83 ± 25.35 µmol TE g^−1^ of compound, FRAP—8341.18 ± 177.72 µM FeSO_4_ g^−1^ compound)	[51]
(6*E*,10*E*,14*E*)-16-hydroxy-2,6,10,14-tetramethylhexadeca-2,6,10,14-tetraen-4-one (21)	Cytotoxicity (against mouse fibroblast cell line (L929), IC_50_ = 18 μg mL^−^^1^)	[30]
(10*E*,14*E*)-16-hydroxy-2,10,14-trimethyl-6-methylenehexadeca-2,10,14-triene-4,7-dione (22)	Antimicrobial activity against gram-positive bacteria (*Bacillus*, MIC = 8 μg mL^−^^1^) and marine fungi (MIC = 8 μg mL^−^^1^) (such as *Corollospora maritima*, *Lulworthia* sp., and *Dendryphiella salina*)	[23]
Eleganolal (23)	Antioxidant potential (ORAC—667.48 ± 10.96 µmol TE g^−1^ of compound, FRAP—8635.37 ± 389.54 µM FeSO_4_ g^−1^ compound)	[51]
(6*E*,14*E*)-16-hydroxy-2,6,14-trimethyl-10-methylenehexadeca-2,6,14-triene-4,11-dione (25)	Antimicrobial activity against gram-positive bacteria (*Bacillus,* MIC = 8 μg mL^−^^1^) and marine fungi (MIC = 8 μg mL^−^^1^) (such as *Corollospora maritima*, *Lulworthia* sp., and *Dendryphiella salina*)	[23]
Eleganolonebutenolide (32)	Cytotoxicity (against mouse fibroblast cell line (L929), IC_50_ = 27 μg mL^−^^1^)	[30]
14,15-dihydro-eleganonic acid (33)	Cytotoxicity (against mouse fibroblast cell line (L929), IC_50_ = 20 μg mL^−^^1^)	[30]
Geranylgeraniol (39)	Antifouling activity—macroalgal spore and zygote development (*Enteromorpha intestinalis* (67.3 ± 2.1% GI); *Ulva lactuca* (83.1% ± 3.4% GI)) and inhibition of adhesion of the blue mussel *Mytilus edulis* (77.0% ± 2.8% IPA)	[23]
Bifurcanol (41)	Antimitotic activity (assay of cytotoxicity activity—inhibition of development of fertilized eggs of the common sea urchin *Paracentrotus lividus*—EC_50_ = 4 μg mL^−^^1^).	[43]
	Cytotoxicity (against mouse fibroblast cell line (L929), IC_50_ = 24 μg mL^−^^1^).	[30]
DCM extract (3.92 ± 0.09% (w/w))	Antioxidant activity (in vitro*:* DPPH assay: IC_50_ = 365.57 ± 10.04 μg mL^−^^1^, ABTS assay: IC_50_ = 116.25 ± 2.54 μg mL^−^^1^)	[38]
	Anti-inflammatory activity (NO production (% of LPS): 6% at 50 μg mL^−^^1^)	
	Antibacterial activity (against both gram-positive (*Staphylococcus aureus* ATCC®6538 (MIC = 1024 μg mL^−^^1^), *Staphylococcus aureus* ATCC®43300 (MIC = 2048 μg mL^−^^1^)) and gram-negative (*Escherichia coli* ATCC®25922 (MIC = 2048 μg mL^−^^1^)) strains)	
	Synergistic effects with antibiotic:Against *S. aureus* ATCC®6538 (Gent (MIC = 32 μg mL^−^^1^); Gent + Ext (MIC =16 μg mL^−^^1^); Tetra (MIC = 16 μg mL^−^^1^); Tetra + Ext (MIC = 8 μg mL^−^^1^))Against *S. aureus* ATCC®43300 (Rif (MIC = 16 μg mL^−^^1^); Rif + Ext (MIC < 2 μg mL^−^^1^); Gent (MIC > 256 μg mL^−^^1^); Gent+Ext (MIC = 16 μg mL^−^^1^); Tetra (MIC > 256 μg mL^−^^1^); Tetra + Ext (MIC < 2 μg mL^−^^1^))Against *E. coli* ATCC®25922 (Rif (MIC = 32 μg mL^−^^1^); Rif + Ext (MIC = 16 μg mL^−^^1^); Gent (MIC > 256 μg mL^−^^1^); Gent+Ext (MIC < 2 μg mL^−^^1^); Tetra (MIC = 18 μg mL^−^^1^); Tetra + Ext (MIC < 2 μg mL^−^^1^))	
EtOAc extract *	Antiprotozoal activity (against erythrocytes infected by a resistant K1 strain of *Plasmodium falciparum* (100% of GI at 9.7 μg mL^−^^1^), *Trypanosoma cruzi* trypamastigostes (78% of GI at 9.7 μg mL^−^^1^), and *Leishmania donovani* amastigotes (100% of GI at 9.7 μg mL^−^^1^), IC_50_ = 3.8 μg mL^−^^1^)	[35]
	Cytotoxicity activity (against L6 cells, rat skeletal myoblasts, IC_50_ = 6 μg mL^−^^1^)	
EtOAc:MeOH (1:1) soluble extract*	Anti-tubercular activity (against *Mycobacterium tuberculosis,* MIC = 64.0 μg mL^−^^1^)	[61]
	Antiprotozoal activity (against *Trypanosoma brucei rhodesiense* (IC_50_ = 1.9 μg mL^−^^1^), *Trypanosoma cruzi* (IC_50_ = 34.7 μg mL^−^^1^), and *Leishmania. donovani* (IC_50_ = 6.4 μg mL^−^^1^))	
	Cytotoxicity (IC_50_ = 32.7 μg mL^−^^1^)	
DCM-MeOH ASE extract*	Antioxidant activity (DPPH assay: EC_50_ = 0.56 ± 0.00 mg mL^−1^, reducing activity: 90.97% at 500 mg L^−1^, *β*-carotene-linoleic acid system assay: 76.13% ± 0.55% of inhibition at 500 mg L^−1^, TPC 0.96% dw)	[8]
	Antitumoral activity (tested with Daudi (Human Burkitt’s lymphoma), K562 (Human chronic myelogenous leukemia) (~40% of viable cells), and Jurkat (Human leukemic T cell lymphoblast) (~20% of viable cells) cells)	
MeOH clean and enrich extract *	Antioxidant activity (TPC: 129.17 ± 0.002 mg GAE g^−1^ ext, ORAC: 3151.35 ± 119.33 μmol TE g^−1^ ext, DPPH: IC_50_ = 58.82 (50.65 − 68.31) μg mL^−^^1^)	[54,55]
	Antimicrobial activity (against *Pseudomonas aeruginosa* ATCC 27853 (11.3 ± 1.5 mm ZI), *Escherichia coli* ATCC 105366 (7.0 ± 0.0 mm ZI)*, Escherichia coli* ATCC 25922 (6.7 ± 0.6 mm ZI)*, Staphylococcus aureus* ATCC 25923 (7.0 ± 0 mm ZI), and *Saccharomyces cerevisiae* ATCC 9763 (IC_50_ = 26.68 μg mL^−^^1^))	
	Antitumor activity: cell proliferation inhibition (tested in 2 in vitro carcinoma models, a human colorectal adeno-carcinoma (Caco-2) (IC_50_ = 437.1 (266.0 − 718.1) μg mL^−^^1^), and a human hepatocellular liver cancer (HepG-2) (IC_50_ = 252.0 (162.0 − 392.2) μg mL^−^^1^))	
DCM extract *	Antioxidant activity (TPC: 43.21 ± 0.043 mg GAE g^−1^ ext, ORAC: 589.98 ± 7.33 μmol TE g^−1^ ext, DPPH: IC_50_ = 344.70 (246.10 − 482.80) μg mL^−1^)	[54,55]
	Antimicrobial activity (against gram-negative bacteria (*Pseudomonas aeruginosa* ATCC 27853 (8.3 ± 1.2 mm ZI), *Escherichia coli* ATCC 105366 (8.3 ± 0.6 mm ZI)), and *Saccharomyces cerevisiae* ATCC 9763 (IC_50_ = 17.06 μg mL^−^^1^))	
	Antitumor activity: cell proliferation inhibition and cytotoxicity (tested in 2 in vitro carcinoma models, a human colorectal adeno-carcinoma (Caco-2) (IC_50_ = 82.31 (54.7 − 123.8) μg mL^−^^1^) (IC_50_ = 90.09 (70.82 − 114.6) μg mL^−^^1^), and a human hepatocellular liver cancer (HepG-2) (IC_50_ = 95.63 (69.66 − 131.3) μg mL^−^^1^) (IC_50_ = 123.9 (95.47 − 160.8) μg mL^−^^1^))	
Et_2_O extract(2.2%–2.9% of dw)	Antifouling activity (against 2 marine bacteria, *Cobetia marina* (MIC between 12.2 ± 0.4 to 26.3 ± 1.3 μg mL^−^^1^) and *Pseudoalteromonas haloplanktis* (MIC between 6.5 ± 0.6 to 15.5 ± 0.8 μg mL^−^^1^)*,* and cypris larvae of the barnacle, *Balanus amphitrite* (EC_50_ = 10 μg mL^−^^1^))	[36]
MeOH clean and enriched extract(6.92% of dw)	Anti-proliferative effect (of well-differentiated pathologic cells, such as a human non–small-cell bronchopulmonary carcinoma line (NSCLC-N6) (IC_50_ = 4 μg mL^−^^1^), by terminal differentiation)	[37]
EtOAc extract(4.80% of dw)	Antiprotozoal activity (against *Trypanosomas brucei rhodesiense* trypomastigotes—crude extracts and the most active fraction)	[21]
Et_2_O extract	Antifouling activity (against *Balanus amphitrite* cyprid (EC_50_ = 0.43 (0.88 − 1.26, conf. lim. 95%) μg mL^−^^1^)	[26]
	Toxicity (against *Balanus amphitrite* nauplius (LC = 0.64 (0.26 − 1.14, conf. lim. 95%) μg mL^−^^1^))	
Et_2_O extract(3.15% of dw)	Antifouling activity toward macroalgal spore and zygote development (*Ulva lactuca* (78.7% ± 2.8% GI)), against diatom growth (*Amphora coffeaformis* (83.9% ± 4.2% GI)*, Phaeodartylum tricornutum* (86.2% ± 3.4% GI)*, Cylindrotheca closterium* (69.2% ± 2.4% GI)), and inhibition of adhesion of the blue mussel *Mytilus edulis* (80.6% ± 2.3% IPA)	[23]
Fraction of DCM extract(0.95% of dw)	Neuroprotective effect (prevent changes in mitochondrial potential (218.10% ± 14.87% of control), reduction of H_2_O_2_ levels production (204.50% ± 15.12% of control), revert neurotoxic effect on cell viability to about 20%–25%).	[51]

ABTS - 2,2’-azino-bis(3-ethylbenzothiazoline-6-sulphonic acid; ASE—accelerated solvent extraction; DCM—dichloromethane; DPPH—2,2-diphenyl-1-picrylhydrazyl assay; EC_50_—efficient concentration of extract to decrease microbial concentration by 50%; Ext—extract; FRAP—ferric reducing antioxidant power; Gent—gentamicin; GI—growth inhibition; IC_50_—inhibitory concentration of the extract required to decrease microbial concentration by 50%; LC—lethal concentration; LPS—lipopolysaccharides; MIC—minimal inhibitory concentration; ORAC—oxygen radical absorbent capacity; Rif—rifampicin; SI—selectivity index; TE—trolox equivalents; Tetra—tetracycline; TPC—total phenolic content; ZI—zone of inhibition; *—linear diterpenes were not identified, however the authors justify theoretically that these components may be responsible for the determined biological activities.

## Supplementary Materials

The following are available online at https://www.mdpi.com/1660-3397/17/10/556/s1, Table S1: MS data of linear diterpenes identified in *B. bifurcata*; Table S2: 1H NMR spectral data for linear diterpenes identified in *B. bifurcata*; Table S3: 13C NMR spectral data of linear diterpenes identified in *B. bifurcata*.
