# Supplementary material for: Current Research on the Bioprospection of Linear Diterpenes from Bifurcaria bifurcata: From Extraction Methodologies to Possible Applications
Source: Mar Drugs. 2019 Sep 28;17(10):556. doi: 10.3390/md17100556 (PMC6835738; doi:10.3390/md17100556)
Supplement: Supplementary file 1 [file marinedrugs-17-00556-s001.pdf]

Supplementary Materials:

Current Research on the Bioprospection of Linear Diterpenes from *Bifurcaria bifurcata*: from Extraction Methodologies to Possible Applications

Adriana C.S. Pais [1], Jorge A. Saraiva [2], Sílvia M. Rocha [2], Armando J.D. Silvestre [1] and Sónia A.O. Santos [1,*]

[1] CICECO-Aveiro Institute of Materials, Chemistry Department, University of Aveiro, 3810-193 Aveiro, Portugal;
[2] QOPNA/LAQV & REQUIMTE, Chemistry Department, University of Aveiro, 3810-193 Aveiro, Portugal;
* Correspondence: santos.sonia@ua.pt; Tel.: +351-234-370-711 (S.A.O.S.)

Table S1 – MS data of linear diterpenes identified in *B. bifurcata*.

Compound	MS data	Ref.
**1**	EIMS (70 eV) *m/z*: 288, 189, 153, 135, 121, 85 and 69.	[1]
**2**	EIMS (70 eV) *m/z* (rel. Int.): 320 (1);<br>HRMS (peak matching): *m/z* 320.2362 calc. for $C_{20}H_{32}O_3$, 320.2351.	[2]
**3**	HRMS: 334.2506 $[M]^+$ (calc. for $C_{21}H_{34}O_3$, 334.2508);<br>EIMS (70 eV) *m/z* (rel. int.): 334 $[M]^+$ (0.1), 316 $[M-H_2O]^+$ (0.2), 265(20), 233(100), 215(0.4), 187(0.7), 137(21), 123(60), 107(19), 93(65), 81(40), 69(58), 55(53).	[3]
**4**	HRMS: 304.2408 $[M]^+$ (calc. for $C_{20}H_{32}O_2$, 304.2402);<br>EIMS (70 eV) *m/z* (rel. Int.): 304 $[M]^+$ (0.02), 286 $[M-H_2O]^+$ (0.90), 235 (6), 217(7), 199(6), 189(3), 159(14), 149(7), 147(9), 135(18), 133(16), 123(20), 109(24), 107(30), 95(100), 93(71), 81(86), 69(68), 55(83).	[3]

**5**	HRMS: 304.2410 $[M]^+$ (calc. for $C_{20}H_{32}O_2$, 304.2402);<br>EIMS (70 eV) *m/z* (rel. Int.): 304 $[M]^+$ (0.05), 286 $[M-H_2O]^+$ (0.16), 235 (34), 217(4), 199(4), 189(3), 159(11), 149(10), 147(8), 135(9), 133(14), 123(20), 109(24), 107(23), 95(55), 93(91), 81(87), 69(72), 55(100).	[3]
**6**	HRMS: 304.2407 $[M-H_2O]^+$ (calc. for $C_{20}H_{32}O_2$, 304.2402);<br>EIMS (70 eV) *m/z* (rel. Int.): 304 $[M-H_2O]^+$ (0.3), 286 $[M-2H_2O]^+$ (0.3), 253(0.5), 137(9), 136(9), 95(1), 81(53), 69(100), 55(9).	[4]
**7**	HRMS: 306.2565 $[M-H_2O]^+$ (calc. for $C_{20}H_{34}O_2$, 306.2559);<br>EIMS (70 eV) *m/z* (rel. Int.): 306 $[M-H_2O]^+$ (0.1), 288 $[M-2H_2O]^+$ (0.5), 237(2), 219(2), 201(4), 135(11), 121(8), 109(11), 95(16), 85(2), 81(100), 71(10), 69(25), 67(9), 55(23), 53(6).	[4]
**8**	HRMS: $C_{20}H_{34}O_3$	[5]
**9**	$[M]^+$ 306 $C_{20}H_{34}O_2$; $[M-H_2O]^+$ 288	[6]
**10**	EIMS (70 eV) *m/z* (rel. int.): 284 $[M-H_2O]^+$ (9), 218 (10), 203 (8), 175 (10), 135 (21), 107 (15), 93 (23), 85 (100), 81 (58), 69 (37), 43 (99).	[7]
**11**	HRMS: 316.2042 $[M-H_2O]^+$ (calc. for $C_{20}H_{28}O_3$, 316.2038);<br>EIMS (70 eV) *m/z* (rel. Int.): 334 $[M]^+$ (0.2), 316 $[M-H_2O]^+$ (0.4), 218 (15), 203 (14), 175 (12), 136 (12), 123 (9), 94 (13), 85 (100), 81 (51), 69 (27).	[7]
**12**	HRMS: 316.2044 $[M-H_2O]^+$ (calc. for $C_{20}H_{28}O_3$, 316.2038);<br>EIMS (70 eV) *m/z* (rel. int,): 316 $[M-H_2O]^+$ (0.4), 207 (4), 135 (6), 123 (5), 109 (7), 107 (7), 93 (12), 85 (100), 69 (19), 68 (27), 67 (15), 55 (14).	[8]
**13**	HRMS: *m/z* 322.2505 $[M]^+$ (calc. for $C_{20}H_{34}O_3$, *m/z* 322.2508);<br>EIMS (70 eV) *m/z* (rel. int.): 322 $[M]^+$ (0.1), 304 $[M-H_2O]^+$ (0.1), 286 $[M-2H_2O]^+$ (0.1), 219 (5), 186 (7),168 (13), 119 (22), 107 (18), 93 (38), 85 (96), 69 (15), 57 (100).	[9]
**14**	HRMS (ESI-TOF) *m/z* 347.2559 $[M+Na]^+$ (calc. for $C_{20}H_{36}O_3Na$, 347.2557).	[10]

**15**	HREIMS $[M+Na]^+$ *m/z* 327.23193 (calc. for $C_{20}H_{32}O_2Na$, 327.22945).	[11]
**16**	$[M]^+$ 320 ($C_{20}H_{32}O_3$) (*m/z* calculated for 320.23511, found 320.2353)	[6]
**17**	MS *m/z*: 300 $[M-18]^+$, 83.0497	[12]
**21**	HRMS: 306.2558 $[M]^+$ (calc. for $C_{20}H_{34}O_2$, 306.2559);<br>EIMS (70 eV) *m/z* (rel. int.): 306 $[M]^+$ (0.4), 288 $[M-H_2O]^+$ (0.9), 211 (3), 188 (6), 135 (12),121 (24), 107 (12), 93 (15), 85 (100), 81 (11), 68 (29), 57 (60), 41 (19).	[8]
**22**	(Acetylated): HRMS: 360.2297 $[M]^+$ (calc. for $C_{22}H_{32}O_4$, 360.2300);<br>EIMS (70 eV) *m/z* (rel. Int.): 360 $[M]^+$ (0.1), 300 $[M-HOAc]^+$ (0.5), 277 (2.9), 217 (4.3), 207 (3.4), 199 (1.5), 151 (20.4), 149 (6.7), 134 (4.6), 123 (3.8), 119(3.2), 107(2.7) 95(5.1), 83(100), 77(2.1), 60(1.0), 55(19.3).	[8]
**26**	HRMS: *m/z* 322.2511 $[M]^+$ (calc. for $C_{20}H_{34}O_3$, *m/z* 322.2508);<br>EIMS (70 eV) *m/z* (rel. int.): 322 $[M]^+$ (0.3), 286 $[M-2H_2O]^+$ (0.3), 253 (0.5), 137 (9), 136 (9), 95 (1), 83 (53), 69 (100), 55 (9).	[9]
**27**	HRMS: *m/z* 320.2353 $[M]^+$ (calc. for $C_{20}H_{32}O_3$, *m/z* 320.2352);<br>EIMS (70 eV) *m/z* (rel. int.): 320 $[M]^+$ (0.4), 302 $[M-H_2O]^+$ (0.1), 289 (0.1), 201 (0.1), 151 (2), 138 (2), 99 (10), 95 (6), 93 (3), 83 (100), 81 (6), 73 (5), 71 (6), 69 (6), 55 (19),43 (34).	[9]
**28**	HRMS: *m/z* 322.2501 $[M]^+$ (calc. for $C_{20}H_{34}O_3$, *m/z* 322.2508);<br>EIMS (70 eV) *m/z* (rel. int.): 322 $[M]^+$ (0.2), 286 $[M-2H_2O]^+$ (0.1), 211 (4), 188 (7), 121 (25), 107 (15), 93 (13), 85 (100), 81 (12), 68 (28), 57 (59).	[9]
**31**	HR(+)ESI-MS: *m/z* calc. for $C_{20}H_{30}O_3Na$, $[M + Na]^+$ 341.2093, found: 341.2092	[13]
**32**	HR(+)ESI-MS: *m/z* calc. for $C_{20}H_{28}O_3Na$, $[M + Na]^+$ 339.1936, found: 339.1933	[13]

**33**	HR(+)ESI-MS: *m/z* calc. for $C_{20}H_{32}O_3Na$, $[M + Na]^+$ 343.2249, found: 343.2264	[13]
**34**	HR(+)ESI-MS: *m/z* calc. for $C_{20}H_{32}O_2Na$, $[M + Na]^+$ 327.2300, found: 327.2298	[13]
**35**	HRESI(+)MS: *m/z* calc. for $C_{21}H_{32}O_3K$, $[M + K]^+$ 371.1983, found: 371.2131	[14]
**36**	HREI(+)MS: *m/z* calc. for $C_{20}H_{26}O_2$, $[M]^+$ 298.1932, found: 298.1928	[14]
**39**	$[M]^+$ *m/z* 290, 69 $[C_5H_9]^+$, 81 $[C_6H_9]^+$, 121 $[C_{11}H_{19}]^+$, 272 $[M\text{-}H_2O]^+$	[15]
**40**	EIMS (70 eV) *m/z* (rel. Int.): 288 (2);<br>HRMS (peak matching) *m/z* 288.2465 calc. for $C_{20}H_{32}O$, 288.2453.	[2]
**41**	HRMS of the TMSi ether: $[M\text{-}TMSiOH]^+$ 360.2855 (calc. for $C_{23}H_{40}SiO$, 360.2848);<br>EIMS (70 eV) *m/z* (rel. Int.): 288 (1) $[M\text{-}H_2O]^+$, 257 (1), 147 (11), 135 (31), 109 (36), 93 (57), 81 (100), 43 (77).	[16]
**42**	HRMS: 288.2450 $[M]^+$ (calc. for $C_{20}H_{32}O$, 288,2453);<br>EIMS (70 eV) *m/z* (rel. int.): 288 $[M]^+$ (0.1), 270 $[M\text{-}H_2O]^+$ (0.4), 255 (0.2), 201(10), 187 (4), 175 (9), 159 (12), 148 (19), 135 (55), 119 (32), 107 (60), 93 (100), 81 (35), 69 (70), 55 (41).	[3]
**44**	HRESI(+)MS *m/z* calc. for $C_{20}H_{34}O_2Na$, $[M + Na]^+$ 329.2457, found: 329.2478.	[14]
**46**	$[M]^+$ *m/z* 278, *m/z* 43 $[C_3H_7]^+$, 57 $[C_4H_9]^+$, 68, 82, 95 $[C_7H_{11}]^+$, 109 and 123 $[C_9H_{15}]^+$	[15]

Calc. – calculated; rel. int – relative intensity;

Table S2 – ^{1}H NMR spectral data for linear diterpenes identified in *B. bifurcata*.[a]

	**1 (360 MHz)**	**2 (360 MHz)**	**3 (200 MHz)**	**4 (200 MHz)**	**5 (200 MHz)**	**6 (400 MHz)**	**7 (400 MHz)**	**8 (200 MHz)**	**9 (360 MHz)**	**9 (200 MHz)**
**H**	$CDCl_3$ [b]	$CDCl_3$ [b]	$CDCl_3$ [b]	$CDCl_3$ [b]	$CDCl_3$ [b]	$CDCl_3$ [b]	$CDCl_3$ [b]	$CDCl_3$ [b]	$CDCl_3$ [b]	C_6D_6 [b]
**1**	4.09 *d* (6.8)			9.88 *d* (8.0)	9.82 *d* (8.0)	4.10 *d* (7.0)	4.12 *d* (6.9)	4.19 *d* (6.8)	4.10 *d* (6.7)	4.11 *d* (6.6)
**2**	5.35 *t* (6.8)	5.66 *s*	5.66 *s*	5.78 *d* (8.0)	5.82 *d* (8.0)	5.39 *t* (7.0)	5.38 *t* (7.0)	5.46 *t* (6.8)	5.42 *t* (6.7)	5.50 *t* (6.6)
**3**										
**4**	1.99 *m*	2.17 *m*	2.15 *m*	2.15 *m*	2.55 *t* (7.3)	1.95 – 2.15 *m*	1.99 *t* (7.0)	2.77 *d* (5.0)	2.00 *m*	2.04 *m*
**5**	2.07 *m*	2.16 *d* (4.5)	2.12 *m*	2.15 *m*	2.11 *m*	1.55 – 1.75 *m*	1.40-1.48 *m*	5.65 *dt* (15.0; 5.0)	2.08 *m*	2.12 *m*
**6**	5.07 *t* (6.8)	5.06 *t* (4.5)	5.09 *t* (7.0)	4.99 *t* (4.0)	5.05 *t* (7.3)	4.01 *t* (7.0)	1.38-1.44 *m*	5.57 *d* (15.0)	5.12 *t* (6.7)	5.22 *t* (6.7)
**7**										
**8**	1.99 *m*	2.00 *m*	2.00 *m*	1.95 *m*	1.98 *m*	1.95-2.15 *m*	1.45-1.50 *m*	1.58 *m*	2.00 *m*	2.04 *m*
**9**	2.07 *m*	2.10 *m*	2.10 *m*	1.95 *m*	2.07 *m*	2.10-2.30 *m*	2.05 *m*	2.08 *m*	2.08 *m*	2.18 *m*
**10**	5.32 *t* (6.8)	5.34 *t* (6.8)	5.36 *t* (7.0)	5.26 *t* (6.3)	5.28 *t* (6.8)	5.36 *t* (7.0)	5.37 *t* (7.0)	5.43 *t* (6.6)	5.18 *t* (6.7)	5.25 *t* (6.7)
**11**										
**12**	3.92 *t* (6.7)	3.96 *dd* (6,7)	3.97 *t* (7.0)	3.87 *t* (6.6)	3.90 *t* (6.8)	3.94 *t* (6.7)	3.94 *t* (6.7)	4.00 *t* (7.0)	2.09 *m*	2.21-2.29 *m*
**13**	2.20 *dd* (6.7,7)	2.2 *ddd* (6,7,7)	2.20 *m*	2.19 *m*	2.18 *m*	2.10-2.30 *m*	2.20-2.28 *m*	2.21 *m*	4.37 *ddd* (8.2; 8.2; 5.3)	4.48 *ddd* (8.2; 8.2; 5.1)
**14**	5.04 *t* (7)	5.06 *t* (7)	5.09 *t* (7.0)	4.99 *t* (6.9)	5.05 *t* (7.0)	5.04 *t* (7.0)	5.05 *t* (7.2)	5.11 *t* (7.0)	5.16 *d* (8.2)	5.32 *d* (8.3)
**15**										
**16**	1.68 *s*	1.70 *s*	1.71 *s*	1.61 *s*	1.65 *s*	1.67 *s*	1.69 *s*	1.75 *s*	1.69 *s*	1.63 *s*
**17**	1.60 *s*	1.59 *s*	1.60 *s*	1.52 *s*	1.55 *s*	1.59 *s*	1.60 *s*	1.66 *s*	1.66 *s*	1.58 *s*
**18**	1.57 *s*	1.60 *s*	1.61 *s*	1.52 *s*	1.55 *s*	1.59 *s*	1.60 *s*	1.64 *s*	1.62 *s*	1.60 *s*
**19**	1.56 *s*	1.62 *s*	1.63 *s*	1.53 *s*	1.57 *s*	4.83-5.01 *s*	1.14 *s*	1.32 *s*	1.57 *s*	1.56 *s*
**20**	1.63 *s*	2.14 *s*	2.16 *s*	2.08 *s*	1.93 *s*	1.64 *s*	1.64 *s*	1.70 *s*	1.64 *s*	1.54 *s*
**21**			3.68 *s*							
**Ref.**	[1]	[2]	[3]	[3]	[3]	[4]	[4]	[5]	[7]	

[a] Chemical shits are given in ppm and coupling constants (*J* in parentheses) in Hz; [b] TMS as internal standard.

Table S2 – Cont. [a]

	10 (200 MHz)	11 (200 MHz)		12 (400 MHz)		13 (400 MHz)	14 (500 MHz)	15 (400 MHz)	15 (600 MHz)	15 (250 MHz)
**H**	$CDCl_3$ [b]	$CDCl_3$ [b]	C_6D_6 [b]	$CDCl_3$ [b]	C_6D_6 [b]	$CDCl_3$ [b]	$CDCl_3$	$CDCl_3$	$CDCl_3$ *	$CDCl_3$ [b]
**1**	7.34 *m*	5.58 *d* (2.4)	4.70 *d* (2.5)	-	-	4.16 *d* (6.8)	4.13 *d* (6.8)	4.11 *d* (6.8)	4.13 *d* (6.5)	4.15 *d* (6.5)
**2**	6.28 *m*	3.80 *t* (2.5)<br>2.82 *ddd* (10.0; 5.0; 2.5)	2.86 *m*<br>2.07 *m*	2.64 *d* (19)<br>2.75 *d* (19)	1.87 *d* (19)<br>2.24 *d* (19)	5.44 *t* (6.8)	5.39 *t* (6.8)	5.36 *t* (6.8)	5.39 *t* (6.5)	5.40 *t* (6.5)
**3**										
**4**	2.47 *t* (7.3)	1.90-1.78 *m*	1.82-1.69 *m*	1.75-1.90 *m*	1.18-1.33 *m*	2.74 *d* (4.8)	2.00 *t* (7.0)	2.04 *t* (8.0)	2.02 *m*	2.04 *m*
**5**	2.24 *m*	2.28 *m*	2.02 *m*	2.09 *m*	1.77 *m*	5.57 *dt* (15.5; 4.8)	1.45 *m*	1.97 *dt* (8.0)	2.09 *m*	2.04 *m*
**6**	5.17 *t* (7.0)	5.16 *t* (6.8)	5.01 *t* (7.0)	5.01 *t* (6.8)	4.93 *m*	5.61 *d* (15.5)	1.40 *m*	5.08 *t* (6.4)	5.10 *t* (6.5)	5.10 *t* (6.5)
**7**										
**8**	2.03 *m*	2.03 *m*	2.02 *m*	2.00 *m*	1.97 *m*	1.60 *m*	1.49 *t* (7.8)	2.04 *t* (8.0)	2.02 *m*	2.04 *m*
**9**	2.12 *m*	2.14 *m*	2.12 *m*	2.09 *m*	2.09 *m*	2.15 *m*	2.07 *dt* (7.0; 7.8)	1.97 *dt* (8.0)	2.09 *m*	2.04 *m*
**10**	5.21 *t* (7.0)	5.21 *t* (6.8)	5.22 *t* (6.7)	5.12 *t* (6.8)	5.21 *t* (6.8)	5.27 *t* (7.0)	5.25 *t* (7.0)	5.20 *t* (6.4)	5.22 *t* (6.5)	5.21 *t* (6.5)
**11**										
**12**	2.13 *m*	2.10 *m*	2.24 *m*	2.07 *m*	2.21 *m*	2.12 *m*	2.11 *d* (6.9)	2.99 *br,s*	3.01 *s*	3.03 *s*
**13**	4.41 *ddd* (8.3; 8.3; 5.0)	4.40 *ddd* (8.2; 8.2; 8.2; 5.3)	4.45 *ddd* (8.3; 8.3; 5.5)	4.33 *ddd* (8.3; 8.3; 5.2)	4.46 *ddd* (8.3; 8.3; 5.5)	4.43 *ddd* (8.2; 8.2; 5.3)	4.41 *dt* (8.2; 6.9)			
**14**	5.16 *d* (8.3)	5.15 *d* (8.2)	5.29 *d* (8.3)	5.07 *d* (8.3)	5.31 *d* (8.3)	5.15 *d* (8.2)	5.13 *d* (8.2)		6.10 *s*	6.10 *s*
**15**										
**16**	1.72 *s*	1.72 *s*	1.61 *s*	1.65 *s*	1.61 *s*	1.72 *s*	1.69 *br.s*	1.55 *br,s*	1.86 *s*	1.87 *s*
**17**	1.69 *s*	1.69 *s*	1.56 *s*	1.62 *s*	1.56 *s*	1.69 *s*	1.66 *br.s*	1.82 *br,s*	2.12 *s*	2.12 *s*
**18**	1.66 *s*	1.66 *s*	1.59 *s*	1.59 *s*	1.59 *s*	1.67 *s*	1.65 *br.s*	1.62 *br,s*	1.59 *s*	1.60 *s*
**19**	1.59 *s*	1.65 *s*	1.47 *s*	1.55 *s*	1.43 *s*	1.28 *s*	1.14 *s*	1.55 *br,s*	1.59 *s*	1.60 *s*
**20**	7.21 *s*			5.36 *s*	4.69 *s*	1.65 *s*	1.65 *br.s*	1.55 *br,s*	1.66 *s*	1.66 *s*
**21**										
**Ref.**	[7]	[7]		[8]		[9]	[10]	[11]	[13]	[8]

[a] Chemical shits are given in ppm and coupling constants (*J* in parentheses) in Hz; [b] TMS as internal standard.

Table S2 – Cont. [a]

	15 (400 MHz)		17	18 (250 MHz)	19 (500 MHz)	20 (500 MHz)	21 (400 MHz)		22 (400 MHz)		23 (400 MHz)
**H**	$CDCl_3$ [b]	$CDCl_3$		CD_3OD	$CDCl_3$	$CDCl_3$	$CDCl_3$ [b]	C_6D_6 [b]	$CDCl_3$ [b]	C_6D_6 [b]	$CDCl_3$
**1**	4.13 *d* (6.5)	4.09 *d* (6.8)	4.08 *d* (6.7)	7.32 *t* (1.7)		5.57 *d* (2.2)	4.15 *d* (6.5)	4.02 *d* (6.5)	4.58 *d* (6.5)	4.59 *d* (6.5)	9.92 *d* (8.0)
**2**	5.39 *t* (6.5)	5.35 *t* (6.8)	5.42 *t* (6.7)	6.24 *d* (1.1)	5.96 *s*	3.78 *t* (2.4)	5.41 *t* (6.5)	5.40 *t* (6.5)	5.34 *t* (6.5)	5.39 *t* (6.5)	5.87 *d* (8.0)
**3**			1.62			2.80 *ddd* (2.5; 4.8; 10.1)					
**4**	2.02 *m*	1.99 *m*	2.72 *d*	2.40 *t* (7.4)	2.42, 2.53 *m*	1.76, 1.88 *m*	1.98 *m*	2.00 *m*	2.05 *m*	1.91 *t* (8.0)	2.22 *m*
**5**	2.09 *m*	2.06 *m*	5.17 *m*	2.20 *q* (7.4)	2.32 *m*	2.26 *m*	2.10 *m*	2.12 *m*	2.10 *m*	2.00 *m*	2.21 *m*
**6**	5.10 *t* (6.5)	5.06 *t* (6.7)	5.44 *d* (15.6)	5.14 *dt* (1.2; 7.1)	5.10 *t* (7)	5.15 *t* (7)	5.11 *t* (6.5)	5.21 *t* (6.5)	5.12 *t* (7.5)	5.06 *t* (8.0)	5.09 *t* (6.2)
**7**			1.27 *s*								
**8**	2.02 *m*	1.98 *m*	3.55 *dd* (6.7, 12)	2.09 *t* (6.5)	2.14 *t* (7)	2.15 *t* (8)	1.98 *m*	2.03 *m*	2.29 *t* (7.5)	2.35 *t* (7.5)	2.02 *m*
**9**	2.09 *m*	2.08 *m*	2.11	2.01 *q* (6.5)	2.04 *q* (7.5)	2.03 *q* (8)	2.10 *m*	2.11 *m*	2.82 *t* (7.5)	2.62 *t* (7.5)	2.12 *m*
**10**	5.22 *t* (6.5)	5.19 *t* (6.8)		5.22 *dt* (1.2; 6.9)	5.12 *t* (7)	5.20 *t* (7)	5.23 *t* (6.5)	5.21 *t* (6.5)			5.22 *t* (6.2)
**11**			1.62								
**12**	3.01 *s*	2.97 *s*	2.98 *s*	2.99 *s*	3.03 *s*	3.02 *s*	3.01 *s*	2.81 *s*	3.40 *s*	3.27 *s*	3.03 *s*
**13**											
**14**	6.10 *s*	6.06 *s*	6.09 *s*	6.14 *t* (1.3)	6.09 *s*	6.09 *s*	2.26 *d* (6.5)	2.01 *d* (6.5)	6.12 *s*	5.88 *s*	6.10 *s*
**15**	-		2.11				2.07 *m*	2.11 *m*			
**16**	1.86 *s*	1.82 *s*	1.87 *s*	1.85 *d* (1.2)	1.86 *s*	1.86 *s*	0.87 *d* (7.0)	0.82 *d* (7.0)	1.89 *s*	1.43 *s*	1.88 *s*
**17**	2.12 *s*	2.11 *s*		2.08 *d* (1.1)	2.12 *s*	2.12 *s*	0.87 *d* (7.0)	0.82 *d* (7.0)	2.12 *s*	2.04 *s*	2.14 *s*
**18**	1.59 *s*	1.56 *s*		1.65 *s*	1.61 *s*	1.61 *s*	1.60 *s*	1.60 *s*	5.84 *s*<br>6.18 *s*	5.39 *s*<br>5.70 *s*	1.61 *s*
**19**	1.59 *s*	1.55 *s*		1.65 *s*	1.61 *s*	1.61 *s*	1.60 *s*	1.54 *s*	1.62 *s*	1.43 *s*	1.61 *s*
**20**	1.66 *s*	1.61 *s*		7.19 *t* (1.7)	5.81 *s*		1.65 *s*	1.49 *s*	1.70 *s*	1.49 *s*	2.17 *s*
**22**									2.07 *s*	1.70 *s*	
**Ref.**	[8]	[17]	[12]		[18]		[8]		[8]		[17]

[a] Chemical shits are given in ppm and coupling constants (*J* in parentheses) in Hz; [b] TMS as internal standard.

Table S2 – Cont. [a]

H	25 (270 MHz)	25 (400 MHz)	26 (400 MHz)	27 (400 MHz)	28 (400 MHz)	29 (400 MHz)	30 (400 MHz)	31 (600 MHz)	32 (600 MHz)	33 (600 MHz)
	$CDCl_3$ [b]	$CDCl_3$ [b]	$CDCl_3$ [b]	$CDCl_3$ [b]	$CDCl_3$ [b]	$CDCl_3$ [b]	$CDCl_3$ [b]	$CDCl_3$	$CDCl_3$	$CDCl_3$
**1**	4.12 *d* (6.5)	4.15 *d* (6.8)	4.15 *d* (6.8)	a: 3.69 *dd* (11.2; 2.7) b: 3.53 *dd* (11.2; 2.7)	4.14 *d* (6.8)	4.11 *d* (6.7)	4.15 *d* (6.8)			
**2**	5.36 *t* (6.5)	5.40 *t* (6.8)	5.41 *t* (6.8)	4.20 *dd* (7.3; 2.7)	5.43 *t* (6.8)	5.40 *t* (6.7)	5.43 *t* (6.8)	5.71 *s*	5.86 *quintet* (1.6)	5.70 *s*
**3**										
**4**	2.29 *t* (7.0)	2.33 *t* (7.0)	2.04 *m*	2.01 *m*	2.05-2.11 *m*	2.08-2.11 *m*	2.73 *d* (5.2)	2.19 *m*	2.48 *t* (7.3)	2.20 *m*
**5**	2.84 *t* (7.0)	2.81 *t* (7.0)	2.12 *m*	2.17 *m*	1.66 *m*	1.61-1.63 *m*	5.57 *dt* (15.4; 5.2)	2.19 *m*	2.32 *m*	2.20 *m*
**6**			5.11 *t* (6.8)	5.12 *t* (7.0)	4.06 *t* (6.4)	4.03 *t* (6.3)	5.62 *d* (15.4)	5.11 *t* (6.0)	5.10 *dt* (1.2; 6.8)	5.10 *t* (6.1)
**7**										
**8**	2.24 *m*	2.31 *t* (7.0)	2.02 *m*	2.02 *m*	2.06-2.18 *m*	2.11-2.14 *m*	1.61 *m*	2.04 *t* (7.8)	2.05 *t* (7.1)	2.04 *t* (7.0)
**9**	2.15 *m*	2.17 *m*	2.14 *m*	2.13 *m*	2.21 *m*	2.24 *m*	2.16 *m*	2.15 *m*	2.16 d (0.9)	2.14 *m*
**10**	5.18 *t* (6.5)	5.22 *t* (6.8)	5.26 *t* (7.0)	5.22 *t* (7.0)	5.24 *t* (6.5)	5.22 *t* (6.3)	5.25 *t* (6.8)	5.24 *t* (6.8)	5.22 *dt* (0.9; 7.1)	5.24 *t* (6.3)
**11**										
**12**	3.01 *s*	3.03 *s*	3.03 *s*	3.02 *s*	3.02 *s*	3.01 *s*	3.03 *s*	3.05 *s*	3.06 *s*	3.03 *s*
**13**										
**14**	6.30 *s*	6.10 *s*	2.61 *s*	6.10 *s*	2.27 *d* (6.7)	6.07 *s*	6.09 *s*	6.12 *s*	6.12 *m*	2.30 *d* (6.9)
**15**					2.11 *m*					2.13 *m*
**16**	1.88 *s*	1.88 *s*	1.23 *s*	1.87 *s*	0.89 *d* (6.5)	1.84 *s*	2.13 *s*	1.89 *s*	1.89 *d* (0.9)	0.91 *d* (6.7)
**17**	2.12 *s*	2.14 *s*	1.23 *s*	2.13 *s*	0.89 *d* (6.5)	2.10 *s*	1.87 *s*	2.16 *s*	2.16 *d* (0.8)	0.91 *d* (6.7)
**18**	1.59 *s*	1.60 *s*	1.62 *s*	1.61 *s*	1.60 *s*	1.59 *s*	1.60 *s*	1.63 *s*	1.64 *s*	1.62 *s*
**19**	5.70 *s* 5.96 *s*	a: 6.00 *s* b: 5.74 *s*	1.60 *s*	1.61 *s*	a: 5.05 *s* b: 4.87 *s*	a: 5.02 *s* b: 4.84 *s*	1.28 *s*	1.63 *s*	1.63 *s*	1.62 *s*
**20**	1.67 *s*	1.69 *s*	1.68 *s*	a: 5.14 *s* b: 4.97 *s*	1.67 *s*	1.65 *s*	1.66 *s*	2.18 *s*	4.75 *d* (1.6)	2.18 *s*
**21**										
**Ref.**	[8]	[9]	[9]	[9]	[9]	[9]	[9]	[13]	[13]	[13]

[a] Chemical shits are given in ppm and coupling constants (*J* in parentheses) in Hz; [b] TMS as internal standard.

Table S2 – Cont. [a]

	**34 (600 MHz)**	**35 (600 MHz)**	**36 (600 MHz)**	**40 (360 MHz)**	**40 (400 MHz)**	**41 (400 MHz)**	**42 (200 MHz)**	**43 (400 MHz)**	**44 (600 MHz)**
**H**	$CDCl_3$	$CDCl_3$	$CDCl_3$	$CDCl_3$ [b]	$CDCl_3$ [b]	$CDCl_3$ [b]	$CDCl_3$ [b]	$CDCl_3$ [b]	$CDCl_3$
**1**	4.18 *s* (6.5)	4.70 *d* (7.2)	7.36 *t* (1.5)	4.10 *d* (6.8)	4.10 *d* (6.8)	4.15 *d* (6.8)	4.15 *d* (7.0)	4.15 *d* (6.8)	4.17 *d* (6.8)
**2**	5.44 *t* (6.5)	5.38 *t* (7.2)	6.30 *s*	5.37 *t* (6.8)	5.37 *t* (6.8)	5.42 *t* (6.8)	5.42 *t* (7.0)	5.41 *t* (7.0)	5.44 *t* (6.8)
**3**									
**4**	2.05 *m*	2.07 *m*	2.47 *t* (7.6)	2.00 *m*	2.0 *m*	2.00 *m*	2.00-2.20 *m*	2.02 *d* (6.5)	2.07 *m*
**5**	2.14 *m*	2.13 *m*	2.26 *q* (7.5)	2.07 *m*	2.07 *m*	2.07 *m*	2.00-2.20 *m*	1.45-1.50 *m*	2.14 *m*
**6**	5.14 *t* (6.3)	5.12 *t* (6.8)	5.19 *t* (7.0)	5.09 *t* (6.8)	5.09 *t* (6.8)	5.11 *t* (6.8)	5.15 *t* (7.0)	1.45-1.50 *m*	5.13 *t* (6.8)
**7**									
**8**	2.04 *m*	2.3 *t* (7.2)	2.04 *t* (7.6)	2.00 *m*	2.0 *m*	2.00 *m*	2.75 *d* (7.0)	1.68 *m*<br>1.80 *m*	2.05 *s*
**9**	1.59 *m*	2.13 *m*	2.13 *q* (7.6)	2.07 *m*	2.07 *m*	2.07 *m*	5.51 *dt* (15.0; 70)	1.78 *m*<br>1.91 *m*	2.14 *m*
**10**	2.20 *m*	5.26 *t* (6.8)	5.23 *t* (6.8)	5.13 *t* (6.8)	5.13 *t* (6.8)	5.13 *t* (6.8)	6.05 *d* (15.0)	4.25 *t* (7.0)	5.13 *t* (6.8)
**11**									
**12**	6.04 *s*	3.05 *s*	3.25 *s*	2.72 *d* (7.2)	2.72 *d* (7.2)	2.67 *d* (5.6)	5.35 *t* (7.0)	5.41 *t* (7.0)	2.04 *s*
**13**				5.58 *dt* (7.2; 15.6)	5.58 *dt* (15.6; 7.2)	5.57 *dt* (15.6; 5.6)	2.81 *t* (7.0)	2.71 *t* (7.0)	2.14 *m*
**14**	6.09 *s*	6.13 *s*	5.89 *s*	6.10 *d* (15.6)	6.10 *d* (15.6)	5.63 *d* (15.6)	5.11 *t* (7.0)	5.10 *t* (7.0)	5.41 *t* (6.9)
**15**									
**16**	1.89 *s*	1.89 *s*	7.09 *s*	4.84 *s*	1.81 *s*	1.32 *s*	1.68 *s*	1.68 *s*	4.01 *s*
**17**	2.16 *s*	2.16 *s*	2.00 *s*	1.81 *s*	4.84 *s*	1.32 *s*	1.64 *s*	1.62 *s*	1.68 *s*
**18**	2.19 *s*	1.63 *s*	1.61 *s*	1.57 *s*	1.57 *s*	1.57 *s*	1.75 *s*	1.62 *s*	1.62 *s*
**19**	1.62 *s*	1.62 *s*	1.62 *s*	1.58 *s*	1.58 *s*	1.60 *s*	1.59 *s*	1.22 *s*	1.62 *s*
**20**	1.62 *s*	1.74 *s*	7.23 *s*	1.65 *s*	1.65 *s*	1.68 *s*	1.68 *s*	1.67 *s*	1.70 *s*
**21**		8.09 *s*							
**Ref.**	[13]	[14]	[14]	[2]	[16]	[16]	[3]	[5]	[14]

[a] Chemical shits are given in ppm and coupling constants (*J* in parentheses) in Hz; [b] TMS as internal standard.

Table S3 – ^{13}C NMR spectral data of linear diterpenes identified in *B. bifurcata*. [a, b, c]

	1 (90.5 MHz)	2 (50 MHz)	3 (50 MHz)	4 (50 MHz)	5 (50 MHz)	6 (100 MHz)	7 (100 MHz)	8 (62.5 MHz)	9	9 (90 MHz)
**C**	$CDCl_3$ [b]	$CDCl_3$ [b]	$CDCl_3$ [b]	$CDCl_3$ [b]	$CDCl_3$ [b]	$CDCl_3$ [b]	$CDCl_3$ [b]	$CDCl_3$ [b]		$CDCl_3$ [b]
**1**	59.21 *t* (106)	171.3 *s*	167.2 - C	191.3 – CH	190.9 – CH	59.2 – CH_2	59.2 – CH_2	59.4 *t*	59.3	59.3 – CH_2
**2**	123.53 *d* (153)	115.2 *d*	115.3 – CH	127.4 – CH	128.6 – CH	123.7 – CH	123.6 – CH	124.2 *d*	123.6	124.3 – CH
**3**	139.09 *s*	162.4 *s*	159.9 – C	163.9 – C	164.0 – C	139.1 – C	139.4 – C	138.4 *s*	135.0	139.4 – C
**4**	39.40 *t* (94)	41.1 *t*	40.8 – CH_2	40.5 – CH_2	34.1 – CH_2	35.5 – CH_2	39.8 – CH_2	42.3 *t*	39.5	39.5 – CH_2
**5**	26.71 *t* (92)	26.1 *t* (4.5)	26.1 – CH_2	26.0 – CH_2	26.8 – CH_2	33.3 – CH_2	21.9 – CH_2	125.3 *d*	25.7	25.8 – CH_2
**6**	123.98 *d* (150)	122.9 *d* (4.5)	123.0 - CH	122.6 – CH	122.2 – CH	74.9 – CH	41.4 – CH_2	138.8 *d*	124.3	123.6 – CH
**7**	134.89 *s*	136.0 *s*	135.9 – C	136.3 – C	137.0 – C	151.3 – C	72.8 – C	72.9 *s*	134.7	134.8 – C
**8**	39.18 *t* (94)	39.2 *t*	39.2 – CH_2	39.2 – CH_2	39.1 – CH_2	30.9 – CH_2	41.2 – CH_2	42.2 *t*	39.4	39.4 – CH_2
**9**	26.00 *t* (94)	25.9 *t*	25.9 – CH_2	25.8 – CH_2	25.8 – CH_2	26.1 – CH	22.1 – CH_2	22.5 *t*	26.2	26.2 – CH_2
**10**	125.80 *d* (151)	125.9 *d* (6.8)	125.8 – CH	125.6 – CH	125.5 – CH	125.6 – CH	126.1 – CH	126.1 *d*	127.5	127.4 – CH
**11**	136.71 *s*	136.7 *s*	136.9 – C	136.9 – C	137.0 – C	137.1 – C	136.9 – C	137.0 *s*	131.6	131.6 – C
**12**	77.14 *d* (109)	77.3 *d* (6.7)	77.2 – CH	77.1 – CH	77.1 – CH	77.1 – CH	77.1 – CH	77.1 *t*	48.2	48.2 – CH_2
**13**	34.17 *t* (96)	34.2 *t* (6.7; 7)	34.2 – CH_2	34.2 – CH_2	32.3 – CH_2	34.1 – CH_2	34.2 – CH_2	34.2 *t*	65.8	65.6 – CH
**14**	120.23 *d* (151)	120.2 *d* (7)	120.3 – CH	120.3 – CH	120.4 – CH	120.1 – CH	120.1 – CH	120.1 *d*	128.4	128.5 – CH
**15**	134.20 *s*	134.4 *s*	134.1 – C	134.3 – C	134.1 – C	134.6 – C	134.7 – C	134.9 *s*	139.3	135.0 – C
**16**	25.76 *q* (70)	25.8 *q*	25.8 – CH_3	25.6 – CH_3	25.0 – CH_3	25.8 – CH_3	25.9 – CH_3	25.9 *q*	26.4	26.4 – CH_3
**17**	17.89 *q* (95)	17.9 *q*	17.9 – CH_3	17.5 – CH_3	17.9 – CH_3	17.9 – CH_3	18.0 – CH_3	18.0 *q*	15.9	18.2 – CH_3
**18**	11.63 *q* (92)	19.1 *q*	11.5 – CH_3	11.6 – CH_3	11.6 – CH_3	11.7 – CH_3	11.7 – CH_3	11.7 *q*	16.2	16.3 – CH_3
**19**	15.92 *q* (96)	16.0 *q*	15.9 – CH_3	16.0 – CH_3	16.0 – CH_3	109.9 – CH_3	26.7 – CH_3	28.2 *q*	16.3	15.9 – CH_3
**20**	16.17 *q* (96)	11.6 *q*	18.7 – CH_3	17.9 – CH_3	25.8 – CH_3	16.2 – CH_3	16.1 – CH_3	16.4 *q*	18.2	16.2 – CH_3
**21**			50.7 – CH_3							
**22**										
**Ref.**	[1]	[2]	[3]	[3]	[3]	[4]	[4]	[5]	[6]	[7]

[a] Chemical shits are given in ppm and coupling constants (*J* in parentheses) in Hz; [b] TMS as internal standard; [c] Multiplicities were obtained with DEPT sequences.

Table S3 – Cont. [a, b, c]

	9 (50 MHz)	10 (50 MHz)	11 (50 MHz)		12 (100 MHz)		13 (100 MHz)	14 (500 MHz)	15 (100, 25, 18 MHz)
**C**	C_6D_6 [b]	$CDCl_3$ [b]	$CDCl_3$ [b]	C_6D_6 [b]	$CDCl_3$ [b]	C_6D_6 [b]	$CDCl_3$ [b]	$CDCl_3$	$CDCl_3$
**1**	59.3 – CH_2	142.5 – CH	77.6 – CH	78.2 – CH	173.5 – C	173.0 – C	59.4 *t*	59.3 – CH_2	58.8
**2**	125.3 – CH	111.1 – CH	53.7 – CH	54.3 – CH	36.1 – CH_2	35.7 – CH_2	124.3 *d*	123.6 – CH	124.5
**3**	137.6 – C	125.0 – C	43.1 - CH	43.8 - CH	62.3 – C	61.8 – C	138.3 *s*	139.5 – C	137.6
**4**	39.9 – CH_2	25.0 – CH_2	26.8 – CH_2	27.9 – CH_2	29.5 – CH_2	29.3 – CH_2	42.3 *t*	39.9 – CH_2	39.6
**5**	26.7 – CH_2	28.4 – CH_2	25.8 – CH_2	26.7 – CH_2	23.1 – CH_2	23.1 – CH_2	125.2 *d*	22.0 – CH_2	26.5
**6**	124.9 – CH	124.2 – CH	123.0 – CH	124.5– CH	122.5 – CH	123.4 – CH	138.9 *d*	41.7 – CH_2	124.4
**7**	134.9 – C	134.9 – C	134.9 – C	134.5 – C	136.9 – C	136.4 – C	73.0 *s*	72.7 – C	134.6
**8**	39.8 – CH_2	39.5 – CH_2	39.5 – CH_2	40.6 – CH_2	39.4 – CH_2	39.7 – CH_2	42.4 *t*	41.3 – CH_2	39.4
**9**	26.5 – CH_2	26.4 – CH_2	26.4 – CH_2	27.5 – CH_2	26.2 – CH_2	26.4 – CH_2	23.1 *t*	22.8 – CH_2	26.8
**10**	128.2 – CH	127.5 – CH	128.2 – CH	129.1 – CH	127.5 – CH	128.6 – CH	127.5 *d*	128.6 – CH	122.9
**11**	132.1 – C	131.7 – C	131.9 – C	133.3 – C	131.9 – C	132.5 – C	131.7 *s*	131.6 – C	129.5
**12**	48.7 – CH_2	48.2 – CH_2	48.2 – CH_2	49.5 – CH_2	48.2 – CH_2	48.7 – CH_2	48.1 *t*	48.1 – CH_2	55.3
**13**	66.5 – CH	65.6 – CH	65.7 – CH	67.2 – CH	65.5 – CH	66.2 – CH	65.9 *d*	65.9 – CH	198.8
**14**	129.1 – CH	128.5 – CH	127.5 – CH	129.9 – CH	129.2 – CH	129.1 – CH	128.6 *d*	127.5 – CH	129.1
**15**	133.5 – C	135.5 – C	137.0 – C	137.4 – C	135.0 – C	133.7 – C	134.6 *s*	134.8 – C	155.1
**16**	25.8 – CH_3	25.8 – CH_3	25.8 – CH_3	26.6 – CH_3	25.8 – CH_3	25.7 – CH_3	25.8 *q*	25.7 – CH_3	27.5
**17**	18.2 – CH_3	18.2 – CH_3	18.2– CH_3	19.0– CH_3	18.2 – CH_3	18.1 – CH_3	18.2 *q*	18.2 – CH_3	20.5
**18**	16.4 – CH_3	16.2 – CH_3	16.2 – CH_3	17.2 – CH_3	16.1 – CH_3	16.3 – CH_3	16.4 *q*	16.1 – CH_3	15.9
**19**	15.9 – CH_3	15.9 – CH_3	16.0 – CH_3	16.7 – CH_3	15.9 – CH_3	15.7 – CH_3	28.5 *q*	26.7 – CH_3	16.2
**20**	16.2 – CH_3	138.9 - CH	175.6 – C	175.3 – C	82.9 – CH	82.6 – CH	16.2 *q*	16.2 – CH_3	17.6
**21**									
**22**									
**Ref.**	[7]	[7]	[7]		[8]		[9]	[10]	[19]

[a] Chemical shits are given in ppm and coupling constants (*J* in parentheses) in Hz; [b] TMS as internal standard; [c] Multiplicities were obtained with DEPT sequences.

Table S3 – Cont. [a, b, c]

C	15 (100 MHz)	15 (150 MHz)	15 (62.5 MHz)	15 (100 MHz)	15 (100 MHz)	16	18 (62.85 MHz)	19 (62.85 MHz)	20 (62.85 MHz)
	$CDCl_3$	$CDCl_3$ *	$CDCl_3$ [b]		$CDCl_3$		CD_3OD	$CDCl_3$	$CDCl_3$
**1**	59.0 – CH_2	59.2	59.4 – CH_2	59.2 – CH_2	59.33	62.2	138.12 - CH	173.09	77.16
**2**	123.0 – CH	123.4	124.4 – CH	123.4 – CH	123.34	60.8	110.09 – CH	117.31	53.01
**3**	139.8 – C	139.4	139.4 – C	139.4 – C	139.58	60.5	124.22 – C	169.51	42.39
**4**	39.8 – CH_2	39.4	39.7 – CH_2	39.4 – CH_2	39.46	37.9	24.04 – CH_2	24.62	24.85
**5**	26.7 – CH_2	26.2	26.4 – CH_2	26.2 – CH_2	26.21	29.0	27.64 – CH_2	27.52	26.20
**6**	122.7 – CH	124.0	124.1 – CH	124.0 – CH	123.92	122.8	122.0 – CH	122.45	122.02
**7**	138.8 – C	135.0	135.3 – C	135.0 – C	135.02	134.9	134.36 – C	136.56	135.64
**8**	39.8 – CH_2	39.3	39.4 – CH_2	39.3 – CH_2	39.29	38.7	38.46 – CH_2	39.02	38.44
**9**	26.4 – CH_2	26.7	26.9 – CH_2	26.7 – CH_2	26.70	26.1	25.74 – CH_2	26.24	25.65
**10**	123.5 – CH	129.1	123.2 – CH	129.1 – CH	129.10	122.3	123.52 – CH	123.02	122.62
**11**	130.9 – C	129.5	129.8 – C	129.5 – C	129.52	128.4	128.66 – CH	128.37	128.86
**12**	55.3 – CH_2	55.3	55.5 – CH_2	55.3 – CH_2	55.35	54.7	54.17 – CH_2	54.86	54.09
**13**	199.5 - C	199.5	199.9 – C	199.5 – C	199.55	198.9	199.71 – C		199.72
**14**	124.7 – CH	122.8	129.7 – CH	122.8 – CH	122.80	129.1	128.46 – CH	128.58	128.32
**15**	155.8 – C	155.6	155.8 – C	155.6 – C	155.68	155.1	155.63 – C	156.05	155.74
**16**	25.1 – CH_3	27.7	27.7 – CH_3	27.7 – CH_3	27.71	27.1	18.99 – CH_3	18.05	18.98
**17**	17.5 – CH_3	20.6	20.7 – CH_3	20.6 – CH_3	20.66	15.4	25.86 – CH_3	25.13	25.87
**18**	17.2 – CH_3	16.3	16.4 – CH_3	16.3 – CH_3	16.36	15.9	14.64 –CH_3	15.99	14.66
**19**	16.9 – CH_3	15.9	16.0 – CH_3	15.9 – CH_3	15.92	16.2	14.28 – CH_3	13.95	14.28
**20**	19.9 – CH_3	16.2	16.0 – CH_3	16.2 – CH_3	16.24	20.1	141.83 - CH	99.47	175.95
**21**									
**22**									
**Ref.**	[11]	[13]	[8]		[17]	[6]		[18]	

[a] Chemical shits are given in ppm and coupling constants (*J* in parentheses) in Hz; [b] TMS as internal standard; [c] Multiplicities were obtained with DEPT sequences.

Table S3 – Cont. [a, b, c]

C	21 (100 MHz)		22 (100 MHz)		23 (100 MHz)	25 (20.1 MHz)	25 (100 MHz)	26 (100 MHz)	27 (100 MHz)	28 (100 MHz)
	$CDCl_3$ [b]	C_6D_6 [b]	$CDCl_3$ [b]	C_6D_6 [b]	$CDCl_3$	$CDCl_3$ [b]	$CDCl_3$ [b]	$CDCl_3$ [b]	$CDCl_3$ [b]	$CDCl_3$ [b]
**1**	59.3 – CH_2	59.3 – CH_2	61.4 – CH_2	61.2 – CH_2	191.32	59.5 – CH_2	59.2 *t*	59.3 *t*	65.6 *t*	59.3 *t*
**2**	123.3 – CH	125.0 – CH	118.3 – CH	119.4 – CH	127.40	124.3 – CH	124.1 *d*	123.4 *d*	75.0 *d*	123.6 *d*
**3**	139.6 – C	137.9 – C	142.1 – C	141.5 – C	163.87	138.7 – C	138.5 *s*	139.5 *s*	148.2 *s*	139.3 *s*
**4**	39.5 – CH_2	39.8 – CH_2	39.4 – CH_2	39.6 – CH_2	40.56	34.1 – CH_2	33.9 *t*	39.5 *t*	32.4 *t*	35.6 *t*
**5**	26.2 – CH_2	26.6 – CH_2	26.1 – CH_2	26.4 – CH_2	25.62	36.3 – CH_2	36.3 *t*	26.2 *t*	26.3 *t*	33.3 *t*
**6**	124.0 – CH	124.7 – CH	124.2 – CH	124.3 – CH	122.64	201.6 – C	201.1 *s*	124.2 *d*	123.8 *d*	74.9 *d*
**7**	135.0 – C	134.9 – C	134.3 – C	134.7 – C	136.30	148.8 – C	148.0 *s*	134.8 *s*	135.3 *s*	151.3 *s*
**8**	39.2 – CH_2	39.7 – CH_2	34.0 – CH_2	34.3 – CH_2	39.28	31.0 – CH_2	30.8 *t*	39.2 *t*	39.2 *t*	30.8 *t*
**9**	26.6 – CH_2	26.9 – CH_2	36.1 – CH_2	36.3 – CH_2	26.68	27.2 – CH_2	27.0 *t*	26.6 *t*	26.6 *t*	26.6 *t*
**10**	129.4 – CH	129.2 – CH	200.7 – C	199.8 – C	128.90	123.3 – CH	128.4 *d*	130.2 *d*	129.0 *d*	129.1 *d*
**11**	129.4 – C	129.6 – C	143.3 – C	144.1 – C	129.75	130.8 – C	130.6 *s*	128.2 *s*	129.5 *s*	129.6 *s*
**12**	54.4 – CH_2	54.4 – CH_2	46.4 – CH_2	46.7 – CH_2	55.34	55.4 – CH_2	55.2 *t*	55.4 *t*	55.3 *t*	54.2 *t*
**13**	209.7 – C	207.3 – C	197.1 – C	196.0 – C	199.49	199.6 – C	199.4 *s*	212.0 *s*	199.7 *s*	209.7 *s*
**14**	50.5 – CH_2	50.4 – CH_2	123.3 – CH	123.7 – CH	122.86	128.6 – CH	122.9 *d*	51.5 *t*	122.8 *d*	50.8 *t*
**15**	24.4 – CH	24.4 – CH	156.4 – C	154.9 – C	155.66	155.8 – C	155.6 *s*	69.6 *s*	155.8 *s*	24.4 *s*
**16**	22.5 – CH_3	22.6 – CH_3	27.7 – CH_3	27.2 – CH_3	27.73	20.8 – CH_3	27.7 *q*	29.2 *q*	27.7 *q*	22.5 *q*
**17**	22.5 – CH_3	22.6 – CH_3	20.8 – CH_2	20.6 – CH_2	20.68	27.7 – CH_3	20.7 *q*	29.2 *q*	20.7 *q*	22.5 *q*
**18**	16.4 – CH_3	16.5 – CH_3	126.9 – CH_2	125.7 – CH_2	16.40	16.5 – CH_3	16.5 *q*	16.4 *q*	16.4 *q*	16.5 *q*
**19**	15.9 – CH_3	16.0 – CH_3	16.1 – CH_3	16.1 – CH_3	16.05	124.3 – CH_2	124.1 *q*	15.9 *q*	16.0 *q*	110.1 *q*
**20**	16.2 – CH_3	16.2 – CH_3	16.5 – CH_3	16.3 – CH_3	17.60	16.5 – CH_3	16.2 *q*	16.3 *q*	110.8 *t*	16.3 *q*
**21**			171.2 – C	170.2 – C						
**22**			21.1 – CH_3	20.6 – CH_3						
**Ref.**	[8]		[8]		[17]	[8]	[9]	[9]	[9]	[9]

[a] Chemical shits are given in ppm and coupling constants (*J* in parentheses) in Hz; [b] TMS as internal standard; [c] Multiplicities were obtained with DEPT sequences.

Table S3 – Cont. [a, b, c]

C	29 (100 MHz)	30 (100 MHz)	31 (150 MHz)	32 (150 MHz)	33 (150 MHz)	34 (150 MHz)	35 (600 MHz)	36 (600 MHz)
	$CDCl_3$ [b]	$CDCl_3$ [b]	$CDCl_3$	$CDCl_3$	$CDCl_3$	$CDCl_3$	$CDCl_3$	$CDCl_3$
**1**	59.2 *t*	59.3 *t*	171.8	174.2	171.9	59.4	60.7	142.6
**2**	123.6 *d*	124.2 *d*	115.2	115.6	115.2	123.3	117.6	111.1
**3**	139.3 *s*	138.2 *s*	163.0	170.3	162.9	139.8	143.2	125.0
**4**	35.6 *t*	42.1 *t*	41.2	28.7	41.2	39.5	39.5	25.0
**5**	33.4 *t*	125.2 *d*	25.9	25.6	25.9	26.3	26.2	28.5
**6**	74.8 *d*	138.7 *d*	122.9	122.1	123.0	124.0	123.7	123.9
**7**	151.3 *s*	72.9 *s*	136.1	137.3	135.9	135.8	135.3	135.6
**8**	30.9 *t*	42.3 *t*	39.3	39.3	39.3	39.4	39.4	39.5
**9**	26.7 *t*	23.1 *t*	26.7	26.6	26.6	25.7	26.8	26.6
**10**	128.8 *d*	129.3 *d*	129.0	128.7	129.0	40.8	129.2	126.6
**11**	130.0 *s*	129.8 *s*	129.7	130.0	129.4	158.1	129.6	131.8
**12**	55.1 *t*	55.1 *t*	55.4	55.3	54.5	125.7	55.5	38.5
**13**	199.8 *s*	199.3 *s*	199.7	199.4	209.9	191.8	199.5	154.5
**14**	122.9 *d*	122.9 *d*	122.8	122.9	50.5	126.3	122.8	108.8
**15**	156.0 *s*	155.9 *s*	155.9	155.8	24.4	154.5	155.7	120.5
**16**	27.7 *q*	27.7 *q*	27.8	27.8	22.6	27.7	27.8	137.7
**17**	20.7 *q*	20.7 *q*	20.7	20.7	22.6	20.7	20.7	9.9
**18**	16.5 *q*	16.4 *q*	16.4	16.5	16.4	19.2	16.4	15.9
**19**	110.0 *t*	28.2 *q*	16.0	16.2	16.0	16.3	16.0	16.1
**20**	16.3 *q*	16.3 *q*	19.2	73.2	19.2	16.4	16.5	138.8
**21**							161.2	
**22**								
**Ref.**	[9]	[9]	[13]	[13]	[13]	[13]	[14]	[14]

[a] Chemical shits are given in ppm and coupling constants (*J* in parentheses) in Hz; [b] TMS as internal standard; [c] Multiplicities were obtained with DEPT sequences.

Table S3 – Cont. [a, b, c]

C	40 (50 MHz)	40 (100.5 MHz)	41 (100.5 MHz)	42 (50 MHz)	43 (100 MHz)	44 (600 MHz)
	$CDCl_3$ [b]	$CDCl_3$ [b]	$CDCl_3$ [b]	$CDCl_3$ [b]	$CDCl_3$ [b]	$CDCl_3$
**1**	59.0 *t* (6.8)	59.0	59.4	59.4 – CH_2	59.4 *t*	59.4
**2**	123.4 *d* (6.8)	123.3	123.4	123.4 – CH	123.3 *d*	123.3
**3**	139.1 *s*	139.1	139.7	139.6 – C	139.9 *s*	139.8
**4**	39.4 *t*	39.4	39.6	39.5 – CH_2	39.9 *t*	39.3
**5**	26.5 *t*	26.5	26.3	26.4 – CH_2	22.6 *t*	26.3
**6**	123.8 *d* (6.8)	123.8	123.9	124.5 – CH	41.0 *t*	123.9
**7**	135.0 *s*	135.0	135.3	134.6 – C	82.8 *s*	134.6
**8**	39.4 *t*	39.4	39.6	43.1 – CH_2	37.2 *t*	39.7
**9**	26.2 *t*	26.2	26.7	125.6 – CH	30.8 *t*	26.6
**10**	125.1 *d* (6.8)	125.1	125.2	135.9 – CH	84.5 *d*	124.5
**11**	133.5 *s*	133.5	133.7	131.8 – C	134.9 *s*	135.3
**12**	42.7 *t* (7.2)	42.7	42.4	129.4 – CH	125.4 *d*	39.6
**13**	128.7 *d* (7.2; 15.6)	128.7	125.4	27.2 – CH_2	26.8 *t*	26.2
**14**	133.7 *d* (15.6)	133.7	139.2	122.5 – CH	122.8 *d*	126.2
**15**	141.9 *s*	141.9	70.7	133.3 – C	131.5 *s*	134.7
**16**	114.3 *t*	18.5	29.8	25.7 – CH_3	25.6 *q*	69.1
**17**	18.5 *q*	114.3	29.8	17.7 – CH_3	17.7 *q*	13.7
**18**	15.9 *q*	15.9	16.0	12.5 – CH_3	11.4 *q*	16.0
**19**	15.8 *q*	15.8	16.0	16.1 – CH_3	26.7 *q*	16.0
**20**	16.1 *q*	16.1	16.3	16.2– CH_3	16.1 *q*	16.3
**21**						
**22**						
**Ref.**	[2]	[16]	[16]	[3]	[5]	[14]

[a] Chemical shits are given in ppm and coupling constants (*J* in parentheses) in Hz; [b] TMS as internal standard; [c] Multiplicities were obtained with DEPT sequences

References

1. Valls R, Banaigs B, Francisco C, Codomier L, Cave A (1986) An acyclic diterpene from the brown alga *Bifurcaria bifurcata*. Phytochemistry 25:751–752
2. Semmak L, Zerzouf A, Valls R, Banaigs B, Jeanty G, Francisco C (1988) Acyclic diterpenes from *Bifurcaria bifurcata*. Phytochemistry 27:2347–2349
3. Culioli G, Daoudi M, Ortalo-magne A, Valls R, Piovetti L (2001) (S)-12-Hydroxygeranylgeraniol-derived diterpenes from the brown alga Bifurcaria bifurcata. Phytochemistry 57:529–535
4. Culioli G, Ortalo-Magné A, Daoudi M, Thomas-Guyon H, Valls R, Piovetti L (2004) Trihydroxylated linear diterpenes from the brown alga *Bifurcaria bifurcata*. Phytochemistry 65:2063–2069
5. El Hattab M, Ben Mesaoud M, Daoudi M, Ortalo-Magné A, Culioli G, Valls R, Piovetti L (2008) Trihydroxylated linear diterpenes from the brown alga *Bifurcaria bifurcata*. Biochem Syst Ecol 36:484–489
6. Biard JF, Verbist JF, Floch R, Letourneux Y (1980) Epoxieleganolone et eleganediol, deux nouveaux diterpenes de *Bifurcaria bifurcata* Ross (Cystoseiracees). Tetrahedron Lett 21:1849–1852
7. Valls R, Piovetti L, Banaigs B, Archavlis A, Pellegrini M (1995) (S)-13-hydroxygeranylgeraniol-derived furanoditerpenes from *Bifurcaria bifurcata*. Phytochemistry 39:145–149
8. Culioli G, Daoudi M, Mesguiche V, Valls R, Piovetti L (1999) Geranylgeraniol-derived diterpenoids from the brown alga *Bifurcaria bifurcata*. Phytochemistry 52:1447–1454
9. Ortalo-Magné A, Culioli G, Valls R, Pucci B, Piovetti L (2005) Polar acyclic diterpenoids from *Bifurcaria bifurcata* (Fucales, Phaeophyta). Phytochemistry 66:2316–2323
10. Smyrniotopoulos V, Merten C, Kaiser M, Tasdemir D (2017) Bifurcatriol, a new antiprotozoal acyclic diterpene from the brown alga *Bifurcaria bifurcata*. Mar Drugs 15:1–10
11. Gallé J, Attitoua B, Kaiser M, Rusig A, Lobstein A, Vonthron-Sénécheau C (2013) Eleganolone , a Diterpene from the French Marine Alga *Bifurcaria bifurcata* Inhibits Growth of the Human Pathogens *Trypanosoma brucei* and *Plasmodium falciparum*. Mar Drugs 11:599–610
12. Combaut G, Piovetti L (1983) A novel acyclic diterpene from the brown alga *Bifurcaria bifurcata*. Phytochemistry 22:1787–1789
13. Göthel Q, Lichte E, Köck M (2012) Further eleganolone-derived diterpenes from the brown alga *Bifurcaria bifurcata*. Tetrahedron Lett 53:1873–1877
14. Göthel Q, Muñoz J, Köck M (2012) Formyleleganolone and bibifuran, two metabolites from the brown alga *Bifurcaria bifurcata*. Phytochem Lett 5:693–695
15. Santos S, Trindade S, Oliveira C, et al (2017) Lipophilic fraction of cultivated *Bifurcaria bifurcata* R. Ross: Detailed composition and *in vitro* prospection of current challenging bioactive properties. Mar Drugs 15:340
16. Valls, R; Banaigs, B; Piovetti, L; Archavlis, A; Artaud J (1993) Linear diterpene with antimitotic activity from the brown alga *Bifurcaria bifurcata*. Phytochemistry 34:1585–1588
17. Silva J, Alves C, Freitas R, Martins A, Pinteus S, Ribeiro J, Gaspar H, Alfonso A, Pedrosa R (2019) Antioxidant and Neuroprotective Potential of the Brown Seaweed Bifurcaria bifurcata in an in vitro Parkinson ′ s Disease Model. Mar Drugs 17:1–16
18. Hougaard L, Anthoni U, Christophersen C, Nielsen PH (1991) Eleganolone derived diterpenes from *Bifurcaria bifurcata*. Phytochemistry 30:3049–3051
19. Biard JF, Verbist JF, Letoumeux Y (1980) Cétols Diterpeniques à Activite Antimicrobienne de *Bifurcaria bifurcata*. J Med Plant Res 40:288–294
